# Large-Scale Phenomic and Genomic Analysis of Brain Asymmetrical Skew

**DOI:** 10.1093/cercor/bhab075

**Published:** 2021-04-09

**Authors:** Xiang-Zhen Kong, Merel Postema, Dick Schijven, Amaia Carrión Castillo, Antonietta Pepe, Fabrice Crivello, Marc Joliot, Bernard Mazoyer, Simon E Fisher, Clyde Francks

**Affiliations:** Language and Genetics Department, Max Planck Institute for Psycholinguistics, Nijmegen 6525 XD, The Netherlands; Department of Psychology and Behavioral Sciences, Zhejiang University, Hangzhou 310028, China; Language and Genetics Department, Max Planck Institute for Psycholinguistics, Nijmegen 6525 XD, The Netherlands; Language and Genetics Department, Max Planck Institute for Psycholinguistics, Nijmegen 6525 XD, The Netherlands; Language and Genetics Department, Max Planck Institute for Psycholinguistics, Nijmegen 6525 XD, The Netherlands; Institut des Maladies Neurodégénératives, UMR5293, Groupe d’Imagerie Neurofonctionnelle, Commissariat à l'énergie atomique et aux énergies alternatives, CNRS, Université de Bordeaux, Bordeaux cedex 33076, France; Institut des Maladies Neurodégénératives, UMR5293, Groupe d’Imagerie Neurofonctionnelle, Commissariat à l'énergie atomique et aux énergies alternatives, CNRS, Université de Bordeaux, Bordeaux cedex 33076, France; Institut des Maladies Neurodégénératives, UMR5293, Groupe d’Imagerie Neurofonctionnelle, Commissariat à l'énergie atomique et aux énergies alternatives, CNRS, Université de Bordeaux, Bordeaux cedex 33076, France; Institut des Maladies Neurodégénératives, UMR5293, Groupe d’Imagerie Neurofonctionnelle, Commissariat à l'énergie atomique et aux énergies alternatives, CNRS, Université de Bordeaux, Bordeaux cedex 33076, France; Language and Genetics Department, Max Planck Institute for Psycholinguistics, Nijmegen 6525 XD, The Netherlands; Donders Institute for Brain, Cognition and Behavior, Radboud University, Nijmegen 6525 EN, The Netherlands; Language and Genetics Department, Max Planck Institute for Psycholinguistics, Nijmegen 6525 XD, The Netherlands; Donders Institute for Brain, Cognition and Behavior, Radboud University, Nijmegen 6525 EN, The Netherlands

**Keywords:** brain asymmetry, brain torque, genetics, handedness, lateralization

## Abstract

The human cerebral hemispheres show a left–right asymmetrical torque pattern, which has been claimed to be absent in chimpanzees. The functional significance and developmental mechanisms are unknown. Here, we carried out the largest-ever analysis of global brain shape asymmetry in magnetic resonance imaging data. Three population datasets were used, UK Biobank (*N* = 39 678), Human Connectome Project (*N* = 1113), and BIL&GIN (*N* = 453). At the population level, there was an anterior and dorsal skew of the right hemisphere, relative to the left. Both skews were associated independently with handedness, and various regional gray and white matter metrics oppositely in the two hemispheres, as well as other variables related to cognitive functions, sociodemographic factors, and physical and mental health. The two skews showed single nucleotide polymorphisms-based heritabilities of 4–13%, but also substantial polygenicity in causal mixture model analysis, and no individually significant loci were found in genome-wide association studies for either skew. There was evidence for a significant genetic correlation between horizontal brain skew and autism, which requires future replication. These results provide the first large-scale description of population-average brain skews and their inter-individual variations, their replicable associations with handedness, and insights into biological and other factors which associate with human brain asymmetry.

## Introduction

A counter-clockwise twist of the whole brain along the anterior–posterior axis, that is, the fronto-occipital torque, has been widely reported in humans since observations in the middle of the 20th century (e.g., Yakovlev and Rakic 1966; LeMay 1976; Weinberger et al. 1982; Zilles et al. 1996; Watkins et al. 2001; Kong et al. 2018; see Toga and Thompson 2003, for a review). This global twisting is manifested by several features, including a more anteriorly protruding right frontal lobe (frontal petalia) and posteriorly protruding left occipital lobe (occipital petalia), a so-called “bending” of the right frontal and left occipital lobes across the midline, and relative increases in the dimensions (e.g., volume and width) of the right frontal and left occipital poles (Toga and Thompson 2003).

Torque has recently been reported to be mostly or wholly absent in our closest living relatives the chimpanzees (Xiang et al. 2018; Xiang et al. 2019; Neubauer et al. 2020), although some studies have reported torque in chimpanzees (Hopkins et al. 2008) and other primates (Balzeau et al. 2011; Fears et al. 2011; Neubauer et al. 2020). There is also evidence for alterations of torque in cognitive and neuropsychiatric disorders, including developmental stuttering (Mock et al. 2012), dyslexia (Pieniadz et al. 1983), schizophrenia (SCZ; Luchins and Meltzer 1983; Crow 1997; Maller et al. 2017; but see Chapple et al. 2004), attention-deficit/hyperactivity disorder (Shaw et al. 2009), and depression (Maller et al. 2014; Fullard et al. 2019). Although the sample sizes were not large in these previous studies (e.g., 37 cases and 44 controls in Maller et al. 2017; 231 cases and 68 controls in Fullard et al. 2019), and further replication is needed, these results suggest that the global brain asymmetry pattern may reflect an optimal organization of the human brain, and deviation from it might serve as a biomarker of brain dysfunction.

Besides torque on the anterior-occipital axis, asymmetry on the dorsal-ventral axis has also been reported, but less consistently or well described. An early study reported that the left hemisphere was shifted dorsally relative to the right (Best 1986), but recent work based on magnetic resonance imaging (MRI) analysis of 91 human brains found the opposite pattern, that is, the left hemisphere shifted significantly downward relative to the right (Xiang et al. 2019). Again, this pattern was reported to be human specific, in comparison to chimpanzees (Xiang et al. 2019). Variation in “vertical” asymmetry has not been linked to behavioral differences, or disorder risk, as far as we are aware. In addition, neither vertical nor horizontal asymmetries have been measured in large-scale population analysis in thousands of people, to assess their averages, variances, or correlations.

It was posited in 1874 that “difference of [brain] structure of necessity implies difference in function” (Jackson 1874). However, it has proven surprisingly difficult to link brain structural asymmetries to lateralized functions (Josse et al. 2003; Bishop 2013; Tzourio-Mazoyer and Mazoyer 2017; Batista-Garcia-Ramo and Fernandez-Verdecia 2018). For example, handedness is one of the most clearly evident functional lateralizations, such that in the general population roughly 90% of people are right-handed, and 10% left-handed (Peters et al. 2006; de Kovel et al. 2019; Papadatou-Pastou et al. 2020). In a series of studies based on X-ray computed tomography, LeMay and colleagues reported that the right occipital lobe was more often wider than the left in left-handers, which was the opposite of that found in right-handers (LeMay 1976, 1977; Galaburda et al. 1978; Le May and Kido 1978). Some researchers have even attempted to use the asymmetrical anatomy of skull endocasts to infer handedness in hominins (Smith 1925; LeMay 1976; Holloway 2015).

However, other investigations of the relationships between handedness and features of global brain asymmetry, including more recent studies with up-to-date methodology, have produced inconsistent or negative findings (Kertesz and Geschwind 1971; Chiu and Damasio 1980; Koff et al. 1986; Narr et al. 2007). It has therefore been noted that handedness and global brain asymmetry might not be associated at all, and in any case, their relationship is clearly far from absolute (Chiu and Damasio 1980; LeMay 1992; Steele 2000; Narr et al. 2007). Several MRI studies of regional structural asymmetries that may partly reflect global asymmetry, such as cortical thickness asymmetry of frontal and occipital regions, have also failed to find associations with handedness (Good et al. 2001; Watkins et al. 2001; Herve et al. 2006; Kong et al. 2018). Overall, the mixed results may reflect differences in many factors, including limited imaging quality in early studies, statistical power related to small sample sizes (Kong et al. 2020b), and potential biases when measuring global asymmetry, perhaps especially for manual approaches. A large-scale survey using high-resolution imaging, and objective analysis, is therefore needed to understand the relevance of global brain asymmetry to handedness.

Population-level, average left–right differences of global brain anatomy suggest a genetic-developmental program that is inherently lateralized (Francks 2015; de Kovel et al. 2017; Ocklenburg et al. 2017; de Kovel et al. 2018; Gunturkun et al. 2020). Torque has been observed in fetal brains by the second trimester of pregnancy (Weinberger et al. 1982), as have other region-specific brain asymmetries (Francks 2015), which further supports a genetic influence. McShane et al. reported left–right differences of occipital petalia and width that were related to ethnic origin, suggesting genetic contributions to variability (McShane et al. 1984). A recent, large-scale population study indicated a torque-like pattern of cortical thickness asymmetry, with frontal regions being generally thicker on the left hemisphere, and occipital regions thicker on the right (Kong et al. 2018). In the same study, twin and/or family-based analysis found heritabilities of up to roughly 20% for some of these regional cortical thickness asymmetry measures, for example in the lateral occipital and rostral middle frontal regions, which again suggests that genetic variability may affect global brain asymmetries. A study of vervet monkeys (Fears et al. 2011) also reported heritabilities of 10–30% for measures of global brain asymmetry, which were methodologically very similar to those used in the present study, that is, based on skewing brain MRI data in order to register to a symmetrical template (see below).

Recent large-scale genome-wide association studies (GWAS) have identified specific loci involved in regional brain asymmetries (Carrion-Castillo et al. 2019a; Le Guen et al. 2020; Sha et al. 2021), and also in left-handedness (de Kovel and Francks 2019; Wiberg et al. 2019; Cuellar-Partida et al. 2020). Microtubule-related genes have been particularly implicated by these studies, which is consistent with a role of the cytoskeleton in setting up cellular chirality during embryonic development of the left–right axis of other organs in other species (Okumura et al. 2008; Tee et al. 2015; Davison et al. 2016; Inaki et al. 2016). However, the specific genes involved in global brain asymmetry remain unknown. Early life factors that are known to influence handedness such as birthweight and multiple birth (Heikkila et al. 2018; de Kovel et al. 2019) could also contribute to global brain asymmetry, but this has not previously been studied in large population data.

Here, we present the largest-ever analysis of global brain asymmetry, in 3 independent datasets: the UK Biobank (*N* = 39 678), Human Connectome Project (HCP, *N* = 1113) and BIL&GIN (*N* = 453, roughly balanced for left/right handedness). First, the 2 components of global brain asymmetry, that is, the horizontal and vertical asymmetry skews, were extracted from brain MRI data for each individual, to capture global left–right differences along the anterior–posterior and dorsal-ventral axes. The population distributions of these 2 measures were examined in the 3 datasets, to clarify the population-level average direction and variance of each asymmetry component. In addition, the reliability of these 2 measures was confirmed using a test–retest dataset from the HCP (30 subjects in the dataset had 2 scans each). Next, we investigated the relationships of horizontal and vertical skews with handedness in each of the datasets. Then, we used extensive phenotypic data in the UK Biobank, including early life factors, sociodemographic factors, regional gray and white matter measures derived from brain MRI, and variables related to cognitive functions and health, to explore other potential correlates of global brain asymmetry. Furthermore, we estimated the heritabilities of the horizontal and vertical skews using genome-wide genotype data in the UK Biobank data, and twin data from the HCP, and also investigated their genetic correlations with other traits and disorders. We also used the UK Biobank data to screen the genome for single nucleotide polymorphisms (SNPs) that associate with the horizontal or vertical brain skew measures, at the single-SNP, gene, and pathway levels.

## Materials and Methods

### Datasets

#### UK Biobank

Data were obtained from the UK Biobank as part of research application 16 066, with Clyde Francks as the principal applicant. This is a general adult population cohort. The data collection in the UK Biobank, including the consent procedure, has been described elsewhere (Sudlow et al. 2015). Informed consent was obtained by the UK Biobank for all participants. For this study, we used data from the February 2020 release of 39 678 participants’ brain T1-weighted MRI data, after bias field correction and brain extraction (i.e., *T1_unbiased_brain.nii.gz*; Alfaro-Almagro et al. 2018). The median age of the 39 678 subjects was 64 years, range 44–82 years, and 20 998 subjects were female. Handedness was assessed based on responses to the question: “Are you right or left-handed?” with 4 response options: “right-handed,” “left-handed,” “use both right and left equally,” and “prefer not to answer.” Those who preferred not to answer were excluded for association analysis of global brain asymmetry with handedness, leaving 35 338 right-handers, 3712 left-handers, and 614 “ambidextrous” with brain asymmetry measures. We also made use of genome-wide genotype data for SNPs as described previously (Bycroft et al. 2018), as well as other phenotypic data, including early life factors, sociodemographic factors, regional gray and white matter measures that had already been derived from the brain imaging images, and variables related to cognitive functions and health. Information on these additional phenotype measures are available via the Data Showcase on the UK Biobank website (https://www.ukbiobank.ac.uk/). Our study made use of imaging-derived phenotypes generated by an image-processing pipeline developed and run on behalf of UK Biobank (Alfaro-Almagro et al. 2018).

#### Human Connectome Project

The HCP comprises 1113 individuals with MRI data (606 females, age range 22–37 years at the time of scanning) of varying ethnicities (https://humanconnectome.org/). The HCP contains 143 monozygotic (MZ) twin pairs and 85 dizygotic (DZ) twin pairs, as well as other pairs of siblings and unrelated individuals. Brain structure images, after bias field correction and brain extraction (i.e., files of type *T1w_acpc_dc_restore_brain.nii.gz*; Glasser et al. 2013) were used for each subject. The strength of hand preference was assessed with the Edinburgh Handedness Inventory (Oldfield 1971), resulting in scores ranging from −100 (strong left-hand preference) to 100 (strong right-hand preference). In addition, 30 HCP subjects had been scanned twice, so that test–retest analysis was possible in these 30 subjects (age ranges from 22 to 35 years at the scanning time; 20 females, 10 males).

#### BIL&GIN

BIL&GIN (Mazoyer et al. 2016; *N* = 453; 232 females, age ranges 18–57 years at the scanning time). A high-resolution T1-weighted MRI image was used for each individual, and brain images after bias field correction and brain extraction (implemented in *FreeSurfer* v5.3; surfer.nmr.mgh.harvard.edu) were used. Unlike the UK Biobank and HCP, which had natural population proportions of right-handers, BIL&GIN participants had been selected to be roughly balanced for handedness (248 right-handers and 205 left-handers), based on responses to the options: “right-handed, left-handed, or forced left-handed.” In addition, the strength of hand preference had been assessed with the Edinburgh Handedness Inventory (Oldfield 1971), resulting in scores ranging from −100 (strong left-hand preference) to 100 (strong right-hand preference).

### Ethics Statement

This study utilized de-identified data from the baseline and imaging assessments of the UK Biobank, a prospective cohort of 500 000 individuals (age 40–69 years) recruited across Great Britain during 2006–2010. The protocol and consent were approved by the UK Biobank’s Research Ethics Committee. Data from the HCP were approved by the Institutional Review Boards associated with that project. The BIL&GIN study was approved by the local ethics committee (CCPRB Basse-Normandie).

### Global Asymmetry Measurement from T1-Weighted Brain Images

**Figure 1:**
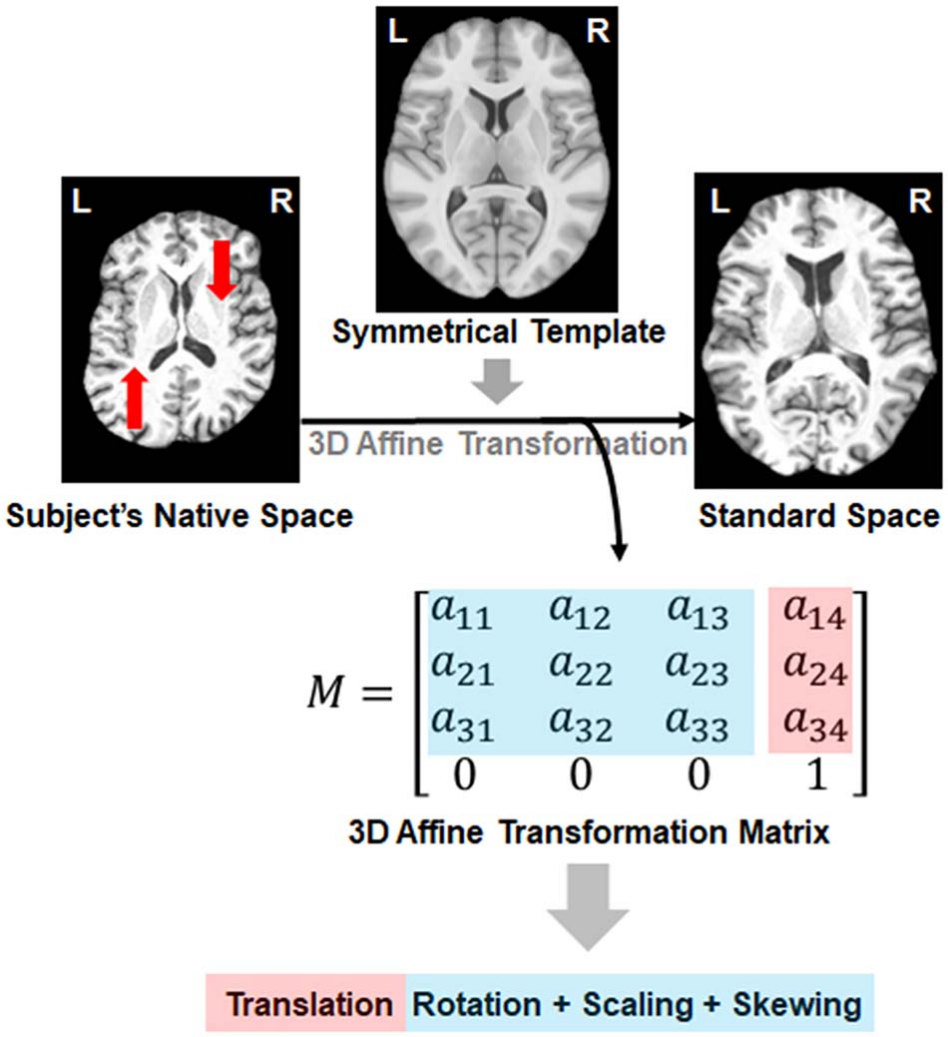
A registration-based approach for estimating global asymmetry skewing. The transformation matrix from the registration procedure captures information about the position alignment (i.e., translation and rotation) as well as scaling, and the amount of skewing during registration. Red arrows indicate the direction in which the native space image is shifted during image registration. Transverse sections are shown, which illustrate the horizontal skew process.

A registration-based approach was used for global asymmetry measurement (Fig. 1), similar to that previously used in a study of vervet monkeys (Fears et al. 2011). Specifically, for each individual participant, an affine transformation was applied to align the T1-weighted brain image (in native space) to the target template image (in the standard MNI space), and an affine transformation matrix was generated as an output. Image processing tools of *flirt* and *avscale* from FSL (version 5.0.10; fsl.fmrib.ox.ac.uk) were used for this analysis. The transformation matrix captures information about global shape differences between individual brain images and the target image, including scaling and skewing with respect to each axis. Although the scaling factors are related to individual brain size, the skewing factors indicate the amount of global twisting to match the template. Note that the registration process is not limited to the cerebral cortex, but is brain-wide (all voxels). In order to measure left–right asymmetries, a left–right symmetrized template was used (i.e., ICBM 2009c Nonlinear Symmetric template). Here, we focused on skewing in the transverse (horizontal) and coronal (vertical) planes, to measure the 2 global asymmetry components, that is, with respect to the frontal-occipital and dorsal-ventral axis, respectively (Fig. 1). A positive horizontal skew is closely akin to typical torque, that is, the protrusions of the right frontal and left occipital regions, which has been reported as the average asymmetry pattern in the human brain (see Introduction). In contrast, a negative horizontal skew indicates a reversal of the typical pattern, with the left frontal and right occipital regions protruding. Similarly, a positive vertical skew indicates an overall twisting downward of the left hemisphere and upward of the right hemisphere, whereas a negative vertical skew indicates the opposite pattern.

### Test–Retest Reliability of Global Asymmetry Measures

The HCP-retest data (30 subjects with 2 scans per subject) allowed us to quantify test–retest reliability of the 2 global asymmetry metrics. Intraclass correlation coefficients (ICC) were calculated using IBM SPSS 20 (Model: Two-Way Mixed; Type: Consistency), where the ICC is conceptualized as the ratio of between-subjects variance to total variance. The ICC is a value between 0 and 1, where 1 indicates perfect reliability (i.e., the within-subject variance is 0).

### Association of Global Brain Asymmetry with Handedness

Association analyses of the horizontal and vertical skew measures were performed for each dataset separately, as per the availability of specific handedness measures and co-variables. In the UK Biobank, the asymmetry differences between handedness groups (−1 = left, 0 = both, and 1 = right, treated as an ordinal variable) were assessed with linear regression models adjusted for sex, age, and nonlinear age (zage^2^, i.e., z-transformed square of the age, i.e., [age-age.mean/age.std]^2^, std=standard deviation), the first 10 principal components (PCs) which capture genome-wide population structure in the genotype data (as made available by the UK Biobank [PC1–10]; Bycroft et al. 2018), and several technical variables related to imaging (Alfaro-Almagro et al. 2018): imaging assessment center (binary), scanner position parameters (continuous *X*/*Y*/*Z*), and signal/contrast-to-noise ratio in T1 (continuous). To explore which handedness group differences mainly contributed to the associations with brain skew measures, we performed 2-sample *t*-tests for each pair of handedness groups (i.e., right-handers vs. left-handers; right-handers vs. both-handers; and both-handers vs. left-handers), separately for each skew measure, after linear adjustment for the confound variables above. To exclude possible outliers in asymmetry measures, we excluded subjects above/below 4 standard deviations from the mean, separately for the horizontal and vertical skew measures. In addition, analyses were repeated when additionally adjusting for brain volume (i.e., gray+white volume) and the brain-size related scaling factors indicated in Figure 1. Python’s *pandas* (pandas.pydata.org), *scipy* (www.scipy.org), and *statsmodels* (www.statsmodels.org) packages were used for these analyses.

In the HCP dataset, asymmetry differences related to the strength of hand preference (ranging from −100 to +100, see above) were examined with linear regression models, adjusting for sex, age, and nonlinear age (zage^2^), “acquisition” (a variable to control for possible scanner status differences across the study period of several years), and “race” (a variable given this label in the HCP data, to capture ethnicity). Analyses were repeated when additionally adjusting for brain volume (“*FS_IntraCranial_Vol*,” intracranial volume estimated with FreeSurfer) and the brain-size related scaling factors indicated in Figure 1. The HCP dataset included twins and siblings, and we therefore used Permutation Analysis of Linear Models (PALM, version alpha111; Winkler et al. 2014) from FSL (version 5.0.10), which has a specialized function for accounting for possible nonindependence caused by family structure. The use of permutation by PALM also means that it is suitable for analyzing the non-normal distribution of manual preference strength. We used 10 000 permutations and calculated 2-tailed *P* values.

In the BIL&GIN dataset, skew differences related to the strength of hand preference (again ranging from −100 to +100, see above) were examined with linear regression models, adjusting for sex, age, and nonlinear age (zage^2^). Given the non-normal distribution of hand preference strength, we further confirmed the association using rank-based Spearman correlation (after adjusting the skew measures for sex, age, and nonlinear age using linear regression). The BIL&GIN dataset also included a binary categorical handedness trait (right- or left-handed) based on a simple questionnaire, and for this trait we tested for brain skew differences between handedness groups using 2-sample *t*-tests, after adjusting the skew measures for sex, age, and nonlinear age. Analyses were repeated when additionally adjusting for brain volume (again intracranial volume estimated with FreeSurfer) and the brain-size related scaling factors indicated in Figure 1. Python’s *pandas* and *statsmodels* packages were used. Although left-handedness was deliberately over-represented in the BIL&GIN dataset (to achieve balance for handedness), we did not attempt to correct for sampling bias, because the findings (see Results) were in line with the other 2 datasets, in which handedness was not over-represented. The relatively small sample size of the BIL&GIN dataset meant that repeat dropping of 9/10 of left-handers to match their population prevalence would make the statistical power too low, and would also result in unequal group sizes (which can create its own statistical issues).

### Phenome-wide Associations of Global Brain Asymmetry

The UK Biobank dataset includes many variables, including early life factors, psychosocial factors, derived imaging traits, and variables related to cognitive functions and health. We ran Phenome-wide Association Scan (pheWAS) analysis for each global asymmetry component, to screen for other associated variables besides handedness and genetic data. We used the package PHEnome Scan Analysis Tool (PHESANT; Millard et al. 2017), which enables comprehensive phenome scans to be performed across all data fields in the UK Biobank. PHESANT uses a rule-based method to automatically determine how to test each variable. The decision rules start by assigning each variable as 1 of 4 types: continuous, ordered categorical, unordered categorical, or binary. A description of PHESANT’s automated rule-based method is given in detail elsewhere (Millard et al. 2017). PHESANT then estimates the bivariate association of an independent variable of interest (in our case either horizontal or vertical brain asymmetry) with each dependent variable in the dataset. Dependent variables with continuous, binary, ordered categorical, and unordered categorical data types, are tested using linear, logistic, ordered logistic, and multinominal logistic regression, respectively. Prior to testing, an inverse normal rank transform is applied to variables of the continuous data type. All analyses were adjusted for covariates as in the handedness association analyses (see above).

We corrected for multiple testing using Bonferroni correction, with a significance threshold determined by dividing 0.05 by the number of tests performed, separately for the horizontal and vertical asymmetry measures as independent variables. We also looked up the results for some variables of particular interest in relation to global brain asymmetry (see Results), which did not necessarily survive multiple testing correction over all phenotypes tested, in which case we report the nominal *P* values. This enables comparison of the identified associations with results for other, possibly related variables.

Finally, as language is a prominently lateralized and human-specific function (Gazzaniga 2009; Price 2012), we ran association analyses between the 2 global asymmetry components and 4 behavioral performance measures related to language, which were available in the HCP dataset. The tasks included were the Penn Word Memory Test, Language Task for functional magnetic resonance imaging, and the NIH Toolbox Oral Reading Recognition Test and Picture Vocabulary Test (Barch et al. 2013). PALM was again used for accounting for family structure in the statistical analyses (see above). Multiple testing was corrected using the Bonferroni method (corrected *P* < 0.05).

### Heritability and Polygenicity Estimation

In the UK Biobank, 550 192 autosomal, directly genotyped SNPs with minor allele frequencies (MAF) > 0.01, genotyping rate > 0.95, and Hardy–Weinberg equilibrium (HWE) *P* > 1 × 10^−6^ were used to build a genetic relationship matrix (GRM) using GCTA (version 1.26.0; Yang et al. 2011). We excluded samples with a genotyping rate of <98% and a kinship coefficient > 0.025 based on this GRM, resulting in a sample size of 30 682. Genome-based restricted maximum likelihood (GREML) analyses using GCTA were performed to estimate the SNP-heritabilities for the horizontal and vertical skew measures, after residualizing for the covariate effects of sex, age, and nonlinear age (zage^2^; see above), the first 10 PCs capturing genome-wide genetic structure (Bycroft et al. 2018), genotyping array, and several technical variables related to imaging as mentioned above. SNP-based heritability is a measure ranging from 0 to 1 that indicates the extent to which variation in a trait is influenced by the combined effects of variations at SNPs distributed over the genome (Vinkhuyzen et al. 2013). Bivariate analyses (Lee et al. 2012) were also run in GCTA, to investigate the SNP-based genetic correlations between the 2 global asymmetry measures, and also with the *x*, *y*, and *z* scaling factors that indicate brain size (see above). Genetic correlation analysis measures the extent to which variability in a pair of traits is influenced by the same genetic variations over the genome.

In addition, we also estimated SNP-based heritability of the 2 global asymmetry measures in the UK Biobank data using GWAS summary statistics for each measure (see below), using LD-score regression as implemented in the LDSC package (v1.0.1) (https://github.com/bulik/ldsc; Finucane et al. 2015). This approach was also used to measure genetic correlation between brain skews and left-handedness, using GWAS summary statistics for left-handedness reported by de Kovel and Francks (2019). Precomputed LD scores from the 1000 Genomes European data (i.e., *eur_w_ld_chr.tar.bz2*) were used, and there was no constraint on the intercept of regression. We also applied causal mixture models to estimate polygenicity (estimated number of causal variants) and discoverability (proportion of phenotypic variance explained on average by a causal variant, σ_β_^2^; Holland et al. 2020), using the MiXeR package (v1.2; https://github.com/precimed/mixer). This analysis was based on the GWAS summary statistics for the brain skew measures as generated in the present study.

Heritability could also be estimated in the HCP dataset, as it included MZ and DZ twin pairs, as well as other siblings and unrelated individuals (see above). We estimated the heritability of each skew measure using variance-component analysis implemented in SOLAR (Almasy and Blangero 1998). Briefly, each skew measure was entered as a dependent variable into separate linear mixed-effects models, which included fixed effects of sex, age, and nonlinear age (zage^2^), and a random effect of genetic similarity, whose covariance structure was determined by the pedigrees. Genetic similarity was coded as 1 for MZ pairs, 0.5 for DZ twin or sibling pairs, and 0 for unrelated pairs of individuals. Maximum likelihood-based bivariate variance decomposition analysis was also applied, again using SOLAR, to estimate the genetic correlation of the 2 skew measures.

### Genome-wide Association Scans

Imputed SNP genotype data (bgen files; imputed data v3—release March 2018) were extracted for the samples with global brain asymmetry measures (*N* = 33 996), and SNP-level statistics were then computed within this set using QCtools (v.2.0.1). We excluded individuals with a mismatch of their self-reported and genetically inferred sex, with putative sex chromosome aneuploidies, or who were outliers based on heterozygosity (heterozygosity > 0.19) and genotype missingness (missing rate > 0.05; Bycroft et al. 2018). We further restricted the analysis to participants with “white British ancestry,” as defined by Bycroft et al. who used a combination of self-report and clustering based on PCs that capture genome-wide diversity in the dataset (“*in.white.British.ancestry.subset*”; Bycroft et al. 2018). We randomly excluded one individual from each pair with a kinship coefficient > 0.0442, as defined within the UK Biobank relatedness file included with the downloaded genotype data. At the SNP level, we excluded SNPs with a minor allele frequency below 1%, Hardy–Weinberg *P* value below 1 × 10^−7^ or imputation quality INFO scores below 0.7 (the latter as provided by the UK Biobank with the imputed data; Bycroft et al. 2018), which resulted in 9 904 141 SNPs genome-wide. GWAS was performed with BGENIE (v.1.2; Bycroft et al. 2018) for each of the residualized global asymmetry measures separately (after accounting for the same covariate effects as for SNP heritability analysis, above), using imputed genotype dosages and an additive model. We applied the commonly-used genome-wide significance threshold *P* value of 5e−08 to assign significance in the context of genome-wide multiple testing, which accounts for the number of SNPs tested in a modern GWAS study, and the correlation structure between SNPs in European ancestry populations (Hoggart et al. 2008; Panagiotou et al. 2012).

### Gene-based and Gene-set Analyses

We derived gene-level association statistics based on the GWAS summary statistics using MAGMA (v1.08; de Leeuw et al. 2015) implemented in FUMA (v1.3.6; Watanabe et al. 2017). In brief, the gene-wise test summarizes the degree of association between a phenotype and SNPs within a given gene (de Leeuw et al. 2015). The gene window was set to 50-kb upstream and downstream to include nearby cis regulatory regions. European samples from the 1000 Genomes phase 3 were used as a reference panel to account for linkage disequilibrium (LD) between SNPs. A significance threshold of *P* < 2.481e−06 (i.e., 0.05/20 151) was applied to correct for multiple testing across all protein coding genes (Ensembl version v92; *n* = 20 151), and a further Bonferroni correction was also considered for having studied 2 skew measures.

The gene-level association statistics were then used to perform gene-set enrichment analysis, again using MAGMA, for gene ontology (GO) terms for biological processes, cellular components, and molecular functions (Ashburner et al. 2000; minimum set size of 10 genes, maximum size 1000 genes, and total *n* = 6576 GO sets meeting these criteria) obtained from the latest MsigDB (v6.2) database (http://software.broadinstitute.org/gsea/msigdb), and Bonferroni correction was performed. This approach tests whether the genes in a given set show, on average, more evidence for association with the trait in question than the rest of the genes in the genome for which scores could be calculated, while accounting for nonindependence of SNPs due to LD.

### Genetic Correlation with Psychiatric Disorders

We calculated genetic correlations of the horizontal and vertical skew measures with 3 psychiatric disorders that have been prominently reported to associate with altered brain asymmetry: ASD (Postema et al. 2019), ADHD (Shaw et al. 2009), and SCZ (Ratnanather et al. 2013; Carrion-Castillo et al. 2019a). This analysis was based on GWAS summary statistics for the brain skew measures as generated in this study, together with publicly available GWAS summary statistics for ASD (18 381 cases and 27 969 controls) (Grove et al. 2019), SCZ (36 989 cases and 113 075 controls) (Schizophrenia Working Group of the Psychiatric Genomics 2014), and ADHD (20 183 cases and 35 191 controls) (Demontis et al. 2019). We used the publicly available summary statistics of the largest-to-date GWAS for each of these disorders. The LDSC package (https://github.com/bulik/ldsc) (Finucane et al. 2015) was used for calculating genetic correlations, and Bonferroni correction was performed for 3 disorders and 2 skews.

### Data and Code Availability

For use of UK Biobank data, application must be made via http://www.ukbiobank.ac.uk/register-apply/. The HCP data are available via https://www.humanconnectome.org/. The BIL&GIN data sharing is based on a collaborative model: http://www.gin.cnrs.fr/BIL&GIN. All analyses were carried out as described in the methods. Software versions and relevant parameters are included in the corresponding methods sections. Scripts are available from the authors upon request.

## Results

### Global Brain Asymmetry in the UK Biobank, HCP, and BIL&GIN Datasets

**Figure 2:**
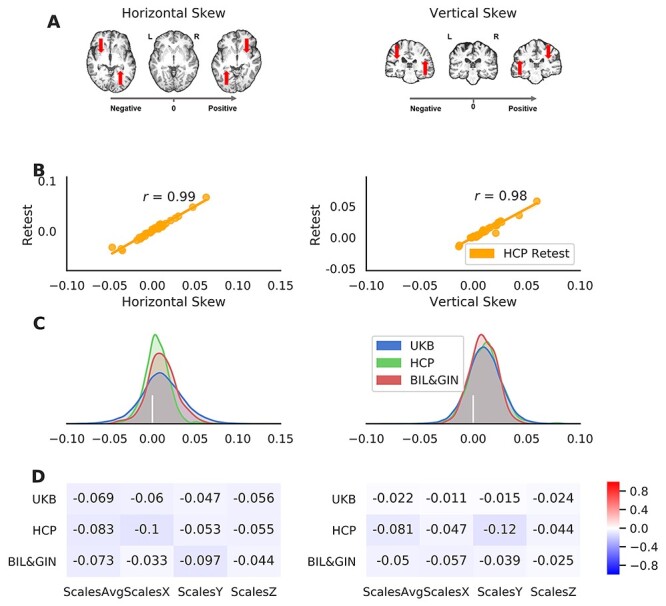
Global brain asymmetry: the horizontal and vertical skews. (*A*) Examples of the human brain with different asymmetry skew scores. The right-pointing gray arrow indicates the axis of the skew scores from negative to positive. The red arrows indicate the skewing needed for each brain during image registration (i.e., registration of the native space brain to a symmetrical template). (*B*) Scatter plots of the asymmetry skews in the HCP-Retest dataset, with the Pearson correlation coefficients. (*C*) Distributions of the asymmetry skew scores in the 3 datasets: UK Biobank in blue, HCP in green, and BIL&GIN in red. The vertical bar in white indicates the position of zero skewing. (*D*) The Pearson correlation coefficients of the asymmetry skew scores with brain size-related scaling factors in the 3-dimensions (ScalesX/ScalesY/ScalesZ) and their average (ScalesAvg).

We extracted 2 global asymmetry components for each individual. A positive score for horizontal skew indicates a global pattern in which the right hemisphere is shifted anteriorly relative to the left, whereas a negative score indicates that the left hemisphere is shifted anteriorly relative to the right (see Fig. 2 and Supplementary Fig. S1 for examples). Note that the skew is calculated as a global feature, not a feature of any particular slice (Supplementary Fig. S1). Similarly, a positive score for vertical skew indicates a global shift downwards of the left hemisphere relative to the right, whereas a negative vertical skew indicates the opposite pattern (Fig. 2 and see Supplementary Fig. S2). Both asymmetry components showed almost perfect reliability (horizontal skew: ICC = 0.989; vertical skew: ICC = 0.977) as indicated in test–retest analysis of data from 30 HCP subjects who underwent 2 scans each (Fig. 2).

The average values of both asymmetry components were positive in the 3 independent datasets (Fig. 2), confirming a population-level pattern of global asymmetry. For horizontal skew, the average pattern involved protrusions of the right frontal and left occipital regions (UK Biobank: *N* = 39 678, Mean = 0.0110, *Std* = 0.025; HCP: *N* = 1113, Mean= 0.0054, *Std* = 0.014; and BIL&GIN: *N* = 453, Mean = 0.011, *Std* = 0.017). One sample *t*-testing indicated a significant difference of each dataset mean from zero (UK Biobank: *t*(39 677) = 86.78, *P* < 5.00e−100, Cohen’s *d* = 0.44; HCP: *t*(1112) = 12.56, *P* = 5.96e−34, Cohen’s *d* = 0.38; and BIL&GIN: *t*(452) = 14.14, *P* = 7.68e−38, Cohen’s *d* = 0.67). The population average horizontal skew matches the widely-observed features of brain torque (e.g., frontal/occipital petalia) in the human brain (see Introduction and e.g., Toga and Thompson 2003).

Regarding the vertical asymmetry skew, the sample means were again all positive (UK Biobank: Mean = 0.0103, *Std* = 0.0158; HCP: Mean  = 0.011, *Std* = 0.015; and BIL&GIN: Mean  = 0.0097, *Std* = 0.012), indicating an average pattern involving downward skewing of the left hemisphere relative to the right. Again the means were significantly different from zero in each dataset (UK Biobank: *t*[39 677] = 131.06, *P* < 5.00e−100, Cohen’s *d* = 0.66; HCP: *t*[1112] = 24.48, *P* < 5.00e−100, Cohen’s *d* = 0.73; and BIL&GIN: *t*[452] = 16.96, *P* = 2.90e−50, Cohen’s *d* = 0.80). Notwithstanding the average asymmetry patterns, the distributions of the vertical and horizontal skews showed considerable individual differences, with for example, 31.8% of participants showing a reversal compared with the average horizontal pattern, and 24.6% showing a reversal compared with the average vertical pattern, in the UK Biobank dataset (Fig. 2). The horizontal and vertical skews showed low correlations with brain-size-related scaling factors in the 3 datasets (Fig. 2): UK Biobank (|*r*|s < 0.07), HCP (|*r*|s < 0.12), and BIL&GIN (|*r*|s < 0.097; see Supplementary Table S1), such that the skews appear to be largely independent of brain size. Also, the 2 skews showed low correlations with each other, and these were inconsistent in strength and direction among the 3 datasets: UK Biobank (*r* = 0.074), HCP (*r* = −0.170), and BIL&GIN (*r* = −0.030).

### Global Brain Asymmetry and Handedness

**Figure 3:**
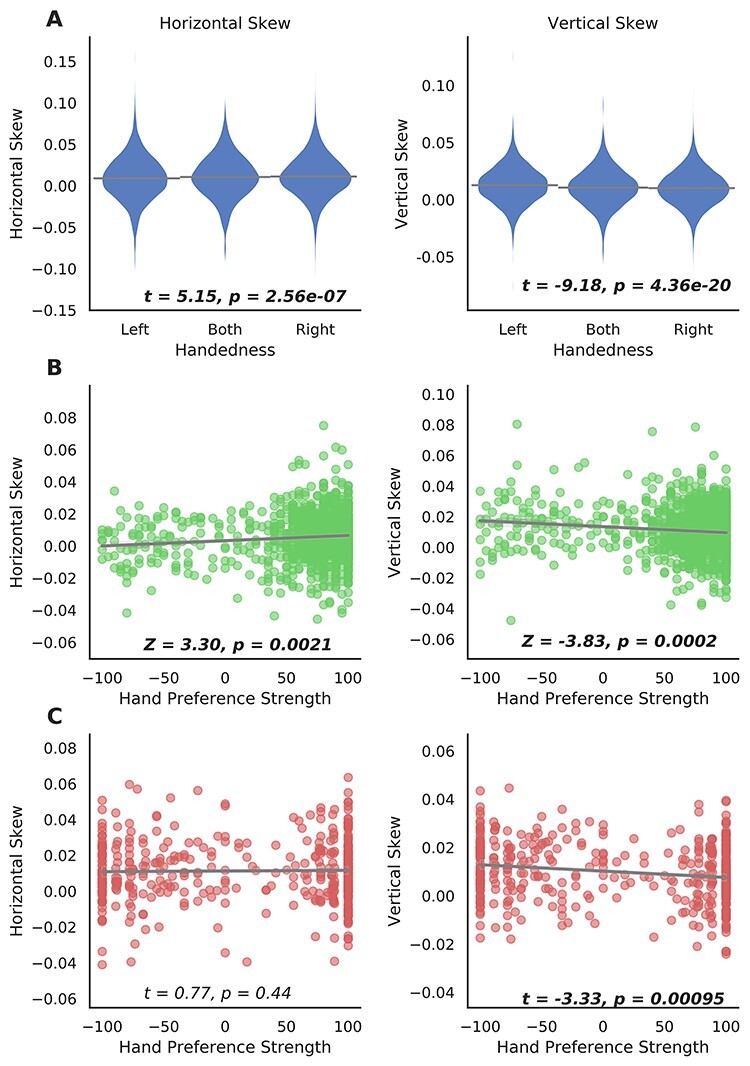
Global asymmetry skews and hand preference. (*A*) Differences in global asymmetry measures between handedness groups in the UK Biobank. (*B*) Scatter plots of global asymmetry measures and hand preference strength in the HCP. (*C*) Scatter plots of global asymmetry measures and hand preference strength in BIL&GIN. Note that the statistical tests of association were based on analyses with covariate effects being controlled for, whereas data are plotted here without adjusting for covariates, for display purposes.

We found significant associations between handedness (left coded as −1, *N* = 3501; ambidextrous coded as 0, *N* = 572 and right coded as 1, *N* = 33 212) and both asymmetry components in the UK Biobank (Fig. 3; horizontal skew: *t* = 5.15, *P* = 2.56e−07 and vertical skew: *t* = −9.18, *P* = 4.36e−20). These associations were mainly contributed by group differences between left-handers and right-handers (horizontal skew: *t* = 5.06, *P* = 4.11e−07, Cohen’s *d* = 0.090; vertical skew: *t* = −9.29, *P* = 1.66e−20; and Cohen’s *d* = 0.165). These results show that right-handers, compared with left-handers, are more likely to show skew along the anterior–posterior axis in the same direction as the population average pattern (i.e., more positive horizontal skew scores). However, on the dorsal-ventral axis, left-handers are more likely to show skew in the same direction as the population average pattern (i.e., more positive vertical skew scores). In addition, a difference was found when comparing vertical skew between left-handers and ambidextrous participants (*t* = −3.24, *P* = 0.0012, and Cohen’s *d* = 0.14), whereas no other comparisons between handedness groups showed significant effects (*P*s > 0.15).

The HCP dataset confirmed that the 2 skews were related to hand preference. Here, a continuous index of the strength of hand preference was available, from −100 (left) to 100 (right), and the analysis accounted for family structure (see Materials and Methods). Again, horizontal skew was positively associated with right-hand preference (*Z* = 3.30, *P* = 0.0021), and vertical skew was positively associated with left-hand preference (*Z* = −3.83, *P* = 0.0002; Fig. 3).

The BIL&GIN dataset further confirmed the finding with respect to vertical skew, that is, this was again positively correlated with increased left-hand preference, *t* = −3.33, *P* = 0.00095 (*rho* = −0.15, *P* = 0.0020) when using the hand preference scale from −100 to 100, and *t* = −3.52, *P* = 0.00048 when using a binary handedness assessment, see Materials and Methods (Left: *N* = 205; Right: *N* = 248). However, in this dataset, the association of hand preference with horizontal skew was not significant, *t* = 0.77, *P* = 0.44 (*rho* = 0.02, *P* = 0.73) for the continuous hand preference scale, and *t* = 0.79, *P* = 0.43 for binary handedness. Nonetheless, the direction of this nonsignificant association in BIL&GIN was consistent with the UK Biobank and HCP datasets. The BIL&GIN dataset provided only 24.4% power to detect the association of horizontal skew with handedness at alpha 0.05, according to the effect size of this association in the UK Biobank (Cohen’s *d* = 0.090). The nonsignificant association between horizontal skew and handedness in BIL&GIN is therefore likely to be a power issue, due to a relatively limited sample size (whereas for the association with vertical skew, BIL&GIN provided 54% power at alpha 0.05, in relation the UK Biobank effect size *d* = 0.165).

When adjusting vertical skew for horizontal skew, and vice versa, the associations of both asymmetry variables with hand preference remained very similar (UK Biobank, 3 handedness groups, horizontal skew, *t* = 5.70, *P* = 1.23e−08; vertical skew, *t* = −9.50, *P* = 2.23e−21; HCP, horizontal skew, *Z* = 2.69, *P* = 0.011; vertical skew, *Z* = −3.32, *P* = 0.0006; and BIL&GIN, hand preference strength, horizontal skew, *t* = 0.67, *P* = 0.50; vertical skew, *t* = −3.57, *P* = 0.00039). This indicates that handedness is primarily associated independently with the 2 asymmetry variables, rather than with any shared variance between them.

In the UK Biobank, there was a significant association between handedness (including left, ambidextrous, and right groups) and one of the brain-size-related scaling factors (i.e., ScalesY: scaling in the anterior–posterior direction, *t* = −3.12, and *P* = 0.00174). However, this association did not replicate in the HCP (*t*s < 1) or BIL&GIN datasets (*t*s < 1 except for ScalesZ: scaling in the superior–inferior direction, *t* = 1.75, and *P* = 0.08). Moreover, when controlling for these scaling factors related to brain size, associations between hand preference and the global asymmetries remained unchanged (UK Biobank, 3 handedness groups, horizontal skew, *t* = 5.17, *P* = 2.32e−07; vertical skew, *t* = −9.21, *P* = 3.57e−20; HCP, horizontal skew, *Z* = 3.38, *P* = 0.0014; vertical skew, *Z* = −3.60, *P* = 0.0004; and BIL&GIN, hand preference strength, horizontal skew, *t* = 0.89, *P* = 0.38; vertical skew, *t* = −3.38, *P* = 0.00079).

### Phenome-wide Associations of Brain Skew Measures

In the UK Biobank dataset, we ran pheWAS to search for other variables associated with the horizontal or vertical components of global brain asymmetry. The variables were those included in our approved UK Biobank project 16 066 “genetics of brain asymmetry and language-related disorders,” and consisted mainly of measures related to cognitive functions, sociodemographics, mental health, physical measures, medical information, and brain imaging measures. In total, the pheWAS analysis included 3562 tests for each of the 2 skew measures. Note that some variables might only be considered as “phenotypes” in a very broad sense, such as country of birth, and home area population density.

**Figure 4:**
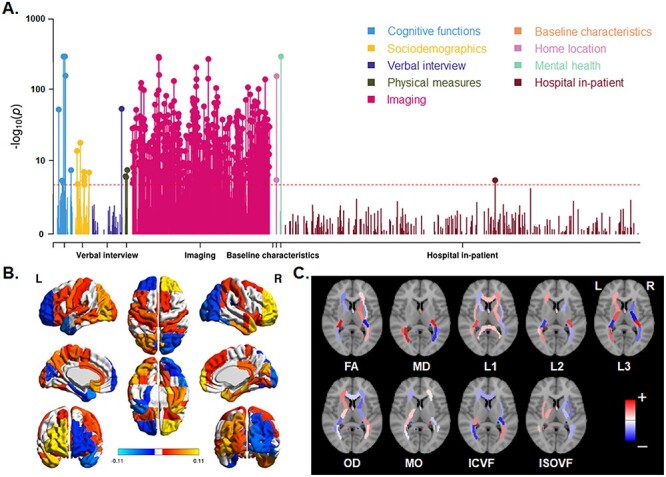
Phenome-wide association analysis for horizontal brain skew. (*A*) Manhattan plots for the associations. Red lines indicate the Bonferroni corrected threshold (*P* < 1.40e−05). (*B*) Significant associations of skew measures with regional gray matter volumes. Red–yellow indicates a positive association; blue indicates a negative association. (*C*) Significant associations of skews with various white matter metrics. Red indicates a positive association; blue indicates a negative association. The per-region names and statistics for parts (*B*) and (*C*) can be found in Supplementary Dataset S1. FA: fractional anisotropy, MD: mean diffusivity, L1/L2/L3: the 3 eigenvalues of diffusion, MO: mode of anisotropy, OD: orientation dispersion, ICVF: intra-axonal volume fraction, ISOVF: isotropic volume fraction.

For each of the 2 skew measures, the pheWAS QQ plot is shown in Supplementary Fig. S3. There were 464 associations below the Bonferroni corrected threshold of 1.40e−05 (0.05/3562) for horizontal skew, and 293 associations for vertical skew. Horizontal skew showed significant associations with variables of various categories, including cognitive functions (e.g., “fluid intelligence score”: *P* = 2.74e−06), sociodemographics (e.g., “age completed full time education”: *P* = 6.19e−08), physical measures (e.g., “body mass index” [BMI]: *P* = 6.05e−07), and mental health (“recent changes in speed/amount of moving or speaking” from the “depression” test: *P* < 1.00e−155; Fig. 4*A* and see Supplementary Dataset S1). The overwhelming majority (440 out of 464) were associations with brain imaging variables (Fig. 4). Interestingly, these latter associations showed a global anterior–posterior “torque” pattern for both gray matter volume (Fig. 4*B*) and white matter metrics (Fig. 4*C*). In terms of gray matter, the volumes of right frontal and left occipital regions positively correlated with horizontal skew, whereas left frontal and right occipital regional volumes negatively correlated with horizontal skew (Fig. 4*B*). Similarly, microstructural measures of homologous white matter tracts showed significant associations with skew measures in opposite directions in the 2 hemispheres (Fig. 4*C*). This confirms that torque manifests not only as a relative shifting of the hemispheres overall, but also as interhemispheric regional asymmetries affecting both gray and white matter (Kong et al. 2018). More details can be seen in Supplementary Figures S5*A*, S6, and Dataset S1.

**Figure 5:**
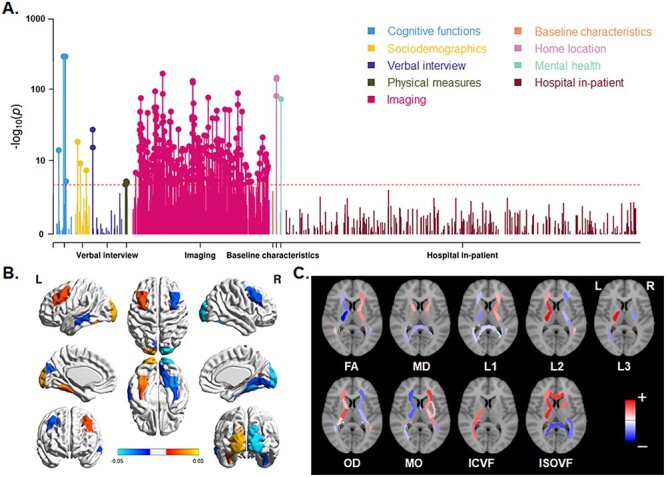
Phenome-wide association analysis for vertical brain skew. (*A*) Manhattan plots for the associations. Red lines indicate the Bonferroni corrected threshold (*P* < 1.40e−05). (*B*) Significant associations of skew measures with regional gray matter volumes. Red–yellow indicates a positive association; blue indicates a negative association. (*C*) Significant associations of skews with various white matter metrics. Red indicates a positive association; blue indicates a negative association. The per-region names and statistics for parts (*B*) and (*C*) can be found in Supplementary Dataset S2. FA: fractional anisotropy, MD: mean diffusivity, L1/L2/L3: the 3 eigenvalues of diffusion, MO: mode of anisotropy, OD: orientation dispersion, ICVF: intra-axonal volume fraction, ISOVF: isotropic volume fraction.

Regarding the vertical skew, we found significant associations with variables of various categories, including cognitive functions (e.g., “time to answer”/prospective memory: *P* = 1.91e−08), sociodemographics (e.g., “transport type for commuting to job workplace: car/motor vehicle”: *P* = 6.19e−08), physical measures (e.g., BMI: *P* = 1.20e−05), and mental health (recent changes in speed/amount of moving or speaking from the depression test: *P* < 4.66e−77; Fig. 5*A* and see Supplementary Dataset S2). Again these significant associations were mostly (274 out of 293) with brain imaging variables. As is shown in Fig. 5*B*, gray matter volumes of the left inferior, medial temporal, and occipital regions correlated positively with vertical skew, whereas the homologous regions in the right hemisphere showed negative correlations. The microstructural metrics of white matter tracts also showed a similar complementary interhemispheric pattern (Fig. 5*C*). More details can be seen in Supplementary Figures S5*B*, S7, and Dataset S2.

Certain early life factors have been shown to influence handedness in the UK Biobank, including birthweight, multiple birth, breastfeeding, and country of birth within the United Kingdom (de Kovel et al. 2019). Maternal smoking was not found to influence handedness in the UK Biobank (de Kovel et al. 2019), although has been implicated in handedness by other studies (e.g., Dragovic et al. 2013). We looked specifically into the pheWAS results for these early life factors, for the 2 brain skew measures. Both skew measures showed significant associations with variables related to place of birth (“country of birth,” *P* = 3.53e−15 for horizontal skew, see Supplementary Dataset S1; “place of birth in United Kingdom—north co-ordinate,” *P* = 2.56e−29 and “place of birth in United Kingdom—east co-ordinate,” *P* = 8.32e−17 for vertical skew, see Supplementary Dataset S2). In addition, breastfeeding (*P* = 0.0030) and birth weight (*P* = 0.025) showed nominally significant associations with vertical skew, whereas other associations were not significant (*P*s > 0.10).

In the HCP dataset, analysis of 4 behavioral performance measures related to language (see Materials and Methods) showed a correlation between horizontal skew and oral reading recognition performance (*Z* = 3.37, permuted *P* = 0.0023, which survived Bonferroni correction for 8 tests, i.e., 2 skew measures times 4 performance measures), such that individuals with more positive horizontal skew showed better oral reading recognition ability. We found no associations with other behavioral measures in the HCP (unadjusted *P*s > 0.10).

### Heritability and Gene Mapping for Brain Skews

In the UK Biobank, significant but low SNP-based heritabilities were found for horizontal skew (*h^2^* = 3.85%, 95% confidence interval [CI] = [0.70%, 7.00%], *P* = 0.024 using GCTA [Yang et al. 2011]; *h^2^* = 4.67%, 95% CI = [0.63%, 8.89%], *P* = 0.029 using LDSC [Finucane et al. 2015]) and vertical skew (*h^2^* = 7.64%, 95% CI = [4.42%, 10.86%], *P* = 3.01e−05 using GCTA [Yang et al. 2011], *h^2^* = 12.95%, 95% CI = [8.94%, 16.96%], *P* = 5.56e−08 using LDSC [Finucane et al. 2015]). In the HCP dataset, which included twins, both skew measures also showed evidence for low heritability: horizontal skew *h^2^* = 9.1%, *P* = 0.041; vertical skew *h^2^* = 10.1%, *P* = 0.030. Vertical skew was slightly more heritable than horizontal skew in both datasets. These findings suggest that genetic variability influences global brain asymmetry, but that most of the variance is not due to genetic variation.

In the UK Biobank data, we also applied causal mixture models (Holland et al. 2020) to estimate the polygenicity (estimated number of causal variants) and discoverability (proportion of phenotypic variance explained on average by each causal variant, σ_β_^2^) for each skew measure. The results indicated that vertical skew has a higher polygenicity (5377 causal variants, AIC = 1.80, and BIC = 12.51) than horizontal skew (1111 causal variants, AIC = 1.92, and BIC = 12.64), at a similar level of discoverability (σ_β_^2^ = 1.47e−05 vs. σ_β_^2^_=_ 2.94e−05).

Genome-wide association analyses showed no significant loci for either horizontal or vertical skew with the standard genome-wide significance threshold of 5e−08 (Hoggart et al. 2008; Panagiotou et al. 2012; see Supplementary Fig. S4), consistent with low heritability and substantial polygenicity. Five SNPs with suggestive association *P* values < 5e−07 are listed in Supplementary Table S2 (one SNP for horizontal skew and 4 for vertical skew), together with their association statistics and nearest genes. Similarly, in gene-based association analysis (de Leeuw et al. 2015), no significant genes were found for either horizontal or vertical skew at the genome-wide, gene-based significance threshold *P* < 2.48e−06 (i.e., 0.05/20151 protein coding genes). The most significant genes were *PLB1* for horizontal skew (chr.2, *Z* = 3.93, and *P* = 4.20e−5) and *PLEC* (chr.8, *Z* = 4.34, and *P* = 7.12e−6), *GRINA* (chr.8, *Z* = 4.24, and *P* = 1.11e−5), and *PARP10* (chr.8, *Z* = 4.08, and *P* = 2.26e−5) for vertical skew.

Gene-set analysis of the GWAS results for vertical skew, using MAGMA (de Leeuw et al. 2015), showed a significant enrichment of association within the GO term “*BP:go_neuron_projection_guidance*” (*beta* = 0.21, *P* = 7.59e−6) after correction for multiple testing (*P* < 7.60e−06, i.e., 0.05/6576 gene sets), which would not be significant with further correction for 2 skews tested. There were no significant sets identified for horizontal skew. Top gene sets with nominal *P* values < 0.001 are listed in Supplementary Table S3.

### Genetic Correlations of Brain Skews with Other Traits

Handedness has shown a low but significant heritability in the UK Biobank in 2 previous studies based on > 330 000 individuals: *h*^2^ = 1.8%, 95% CI = (1.79%, 1.81%; de Kovel and Francks 2019); *h*^2^ = 1.2%, 95% CI = (1.197%, 1.202%; de Kovel et al. 2019; Wiberg et al. 2019). In another study based on several datasets including the UK Biobank, the SNP heritability of handedness was reported to be between 3% and 6% (Cuellar-Partida et al. 2020). (Note that the sample sizes for those studies were much larger than the present study, because the present study is limited to participants who also have brain MRI data available). Using LD score regression with the GWAS summary statistics for handedness (*N* > 330 000) from de Kovel and Francks (2019) and the summary statistics from our GWAS for brain skew measures, we found no significant genetic correlations of hand preference with the skews (*P*s > 0.10). GCTA-based genetic correlation analysis within those individuals having both handedness and brain imaging data (*N* = 32 774) showed similar results (*P*s > 0.10). This was also the case using twin-based co-heritability analysis in the HCP dataset, that is, no significant genetic correlations of handedness and brain skews (*P*s > 0.10).

Brain size-related scaling factors showed high heritabilities in both the UK Biobank (ScalesAvg: *h*^2^ = 72.11%; ScalesX: *h*^2^ = 56.35%; ScalesY: *h*^2^ = 46.28%; and ScalesZ: *h*^2^ = 60.36%) and the HCP (ScalesAvg: *h*^2^ = 92.1%; ScalesX: *h*^2^ = 85.5%; ScalesY: *h*^2^ = 85.0%; and ScalesZ: *h*^2^ = 86.6%), which was expected because of the high heritability of brain size (UK Biobank: *h*^2^ = 72.3%; HCP: *h*^2^ = 87.8%). There were no significant genetic correlations between these scaling factors and brain skew measures (*P*s > 0.10; again using GCTA in the UK Biobank and twin-based co-heritability analysis in the HCP), except for a consistent negative genetic correlation between horizontal skew and ScalesX (scaling in the left–right axis; UK Biobank: *r_g_* = −0.212, *P* = 0.0343; HCP: *r_g_* = −0.291, *P* = 0.0341, *P* values not adjusted for multiple testing). Thus, some of the same genetic variability that contributes to a wider brain may also contribute to a more positive horizontal skew (e.g., anterior shift of the right hemisphere).

In addition, within the UK Biobank data, we ran genetic correlation analyses for brain skews in relation to traits that showed significant associations in the pheWAS analysis, using GCTA. Eight genetic correlations were nominally significant (*P* < 0.01), including gray matter volume in the left Heschl’s Gyrus (*Z* = 2.36 and *P* = 0.0093) with horizontal skew, mean MO (mode of anisotropy) in the left uncinate fasciculus (*Z* = −2.40 and *P* = 0.0083) with horizontal skew, and place of birth in United Kingdom—east co-ordinate (*Z* = −2.42 and *P* = 0.0078) with vertical skew, but none survived multiple testing correction (see Supplementary Table S4). Although horizontal skew showed a significant correlation with oral reading recognition in the HCP data (see above), no significant genetic correlation was observed between them (*P* = 0.54), using SOLAR (Almasy and Blangero 1998).

Using the most recent GWAS summary statistics for 3 psychiatric disorders that have been proposed to associate with altered brain asymmetry (Postema et al. 2019; Carrion-Castillo et al. 2019a), that is, autism spectrum disorder (ASD; Grove et al. 2019), attention-deficit/hyperctivity disorder (ADHD; Demontis et al. 2019), and (SCZ; Schizophrenia Working Group of the Psychiatric Genomics, 2014), we found that horizontal skew showed evidence for genetic correlation (LDSC package; Finucane et al. 2015) with ASD (*r*_rg_ = −0.40, *P* = 0.0075, significant at *P* < 0.05 after Bonferroni correction for 3 disorders and 2 skews), while the genetic correlation of ASD with vertical skew was weaker (*r*_rg_ = −0.17 and *P* = 0.069). Other genetic correlations were not significant (*P*s > 0.10).

## Discussion

We carried out the largest-ever analysis of global brain shape asymmetry, that is, the horizontal and vertical asymmetry skews, in 3 independent datasets. The largest of the datasets comprised over 39 000 participants. At the population level, there was an anterior and dorsal skew of the right hemisphere, relative to the left. The population variances of these skews were largely independent, but both showed replicable associations with handedness, which establishes a link between lateralized structure and function of the human brain. The 2 skews also showed associations with multiple regional gray and white matter metrics, as well as various phenotypic variables including cognitive functions, sociodemographic physical, and mental health measures. The 2 skews showed SNP-based heritabilities of 4–13% depending on the method used to assess this, but no significant loci were found in GWASs, probably due to substantial polygenicity together with relatively low heritability. There was evidence for a significant genetic correlation between horizontal skew and ASD, which may be consistent with the subtle but widespread alterations of cortical regional asymmetries observed in ASD in a recent large-scale study (Postema et al. 2019), and a genetic overlap between multivariate brain asymmetry and ASD reported in another recent study of the UK Biobank data (Sha et al. 2021). Future replication of the genetic correlation between brain skew and ASD will be needed when independent data are available on a comparably large scale.

### Global Brain Asymmetry

We measured the anterior–posterior and superior–inferior aspects of global left–right asymmetry in the human brain, using 3 population datasets and an automated, registration-based approach. The approach contrasts with older, manual methods for evaluating global asymmetry, or approaches using regionally restricted frontal and/or occipital hemispheric differences as proxies for overall torque (e.g., LeMay 1976; Bear et al. 1986; Barrick et al. 2005; Van Essen et al. 2012; Maller et al. 2014; Fullard et al. 2019). Rather, the registration-based approach of the present study allowed an automatic and objective assessment of global asymmetry based on skewing transformation of the brain as a whole. This provides a truly global measure of left–right asymmetry in the human brain. Moreover, the skew metrics in the present study showed high test–retest reliability in twice-scanned individuals.

We found population-level asymmetrical skews on both the horizontal and vertical axes. The average horizontal skew pattern was consistent with previously-observed features of brain torque in the human brain, involving a more anteriorly protruding right frontal lobe and posteriorly protruding left occipital lobe (i.e., frontal/occipital petalia), and relative increases in the dimensions (e.g., volume and width) of the right frontal and left occipital poles (see Toga and Thompson 2003, for a review). The horizontal skew may also relate to a population-level, frontal-occipital asymmetry gradient in regional cortical thickness, recently reported in a large-scale study (Kong et al. 2018). Indeed, we also observed in the present study that individual differences in horizontal skew showed positive correlations with gray matter volumes of right frontal and left occipital regions, and negative correlations with left frontal and right occipital regions, again in line with previously described features of brain torque (Toga and Thompson 2003).

The mean population-level asymmetry pattern in the vertical plane, that is, along the inferior–superior axis, involved an overall twisting of the left hemisphere downward, and right hemisphere upward. This aspect of global brain asymmetry has not been described consistently in the literature, but we found it to be replicable in the 3 independent cohorts of this study. Our findings are also consistent with another recent report that the left-occipital pole is shifted significantly downward relative to the right, on average (Xiang et al. 2019). We found that individual differences in vertical skew correlated positively with gray matter volumes of left inferior temporal regions and the occipital pole, whereas correlating negatively with the homologous regions of the right hemisphere.

As both vertical and horizontal skews showed associations with numerous, widely-distributed regional gray and white matter metrics, then we can conclude that global asymmetry is not simply a spatial displacement of the left and right hemispheres with respect to one another. Rather, global asymmetry relates to structural differences between the 2 hemispheres, affecting many structures from front to back, and top to bottom, and thus likely relates to functionally meaningful hemispheric differences. Given that global asymmetry measures correlated with both regional gray matter volumes and white matter properties, our analysis does not support a previous suggestion that brain torque is driven by white but not gray matter (Allen et al. 2003). In addition, how much of the total variance of the global asymmetry could be explained by these regional measures together would be an interesting question to pursue in future studies, for example through machine learning with feature selection and cross-validation approaches.

An aspect that was inconsistent across datasets in the present study was the relationship between individual differences in the horizontal and vertical components of global asymmetry. Specifically, a positive correlation between horizontal and vertical skew of 0.074 was found in the UK Biobank, but a negative correlation of similar magnitude was observed in the HCP, and no significant correlation was present in the BIL&GIN. Given that the UK Biobank was by far the largest of the 3 datasets, a low, positive correlation between the vertical and horizontal skews is likely to be the true population pattern. Regardless, it is clear that the 2 components of global brain asymmetry are largely independent in their variabilities. Therefore, consideration of these 2 distinct aspects of global brain asymmetry will be important in future studies of their functional significances and phenotypic associations. In general, our results are consistent with other literature which indicate that multiple different asymmetries of the brain can vary largely independently of each other (Liu et al. 2009; Renteria 2012; Mazoyer et al. 2014; Kong et al. 2018).

### Global Brain Asymmetry and Handedness

We found significant associations of both horizontal and vertical skews with handedness, or the strength of hand preference. On average, left-handers showed relatively lower horizontal asymmetry scores than right-handers, that is, reduced asymmetry along the anterior–posterior axis, and higher vertical asymmetry scores, that is, increased asymmetry along the dorsal-ventral axis. The effect sizes were small, for example, in the UK Biobank: Cohen’s *d* = 0.09 for the handedness association with horizontal skew, and *d* = 0.17 for vertical skew. However, particularly in the large UK Biobank dataset, the significance levels were unambiguous despite the subtle effects (*P* value as low as 5.00e−20 for handedness with vertical skew).

As regards the horizontal component, previous results with regard to handedness have been mixed, with some studies finding an association of handedness with brain torque (LeMay 1976, 1977; Galaburda et al. 1978; Le May and Kido 1978), and others not (Kertesz and Geschwind 1971;Chiu and Damasio 1980 ; Koff et al. 1986 ; Narr et al. 2007). All 3 of our datasets showed the same direction of effect for the association between handedness and horizontal skew, although limited statistical power to detect this effect in the BIL&GIN dataset (*N* = 453) is likely to explain that the association was not significant in this specific dataset. For the vertical skew, the association with handedness was again consistent in direction across all 3 datasets, and also significant in all 3 datasets. The association of handedness with the vertical component of global brain asymmetry is a novel finding, as far as we are aware. Given the small effect sizes in our study, it is clear that inferring the handedness of individuals from their global brain asymmetry is unlikely to be possible. Anatomists and anthropologists have long noted a potential link between left-handedness and brain asymmetry, which was initially considered to involve localized thinning and protrusions of the skull, such that attempts have even been made to use skull endocasts to infer the evolution of handedness in hominins (Smith 1925; LeMay 1976, 1992; Holloway 2015). For example, based on their asymmetrical shapes, a skull from Gibraltar and the “Peking man” were suggested to be from right-handed individuals, whereas a skull from London was suggested to have been from a left-handed individual (for a review, see (LeMay 1992). Unfortunately, relationships between brain structural and functional asymmetries are clearly far from absolute, as evidenced by the present study, as well as others (e.g., Chiu and Damasio 1980; Koff et al. 1986; Narr et al. 2007).

We found no significant genetic correlations between either aspect of global brain asymmetry and handedness in the UK Biobank or HCP datasets. This suggests that genetic factors influencing global brain asymmetry are largely dissociable from those affecting handedness, and therefore that environmental factors, such as early life experiences, may play a more predominant role in causing the associations of handedness with global brain asymmetry measures.

Note that re-handed individuals could be present in the UK Biobank, as it comprises adults with average age over 60 years. No explicit data were collected on re-handing. However, we have previously reported how the rate of left-handedness decreases with increased age in the UK Biobank, which likely relates to more frequent re-handing in those born earlier (de Kovel et al. 2019). In the present study we controlled for age (linear and nonlinear) as a confound factor when testing associations of brain skews with handedness, which will have gone some way to control for re-handing. Reassuringly, we found that the associations between brain skews and handedness were also consistent in independent datasets of younger adults.

### Development of Global Brain Asymmetry

Thus far we know little about the developmental mechanisms which lead to brain asymmetry. Best (1986) proposed a 3D lateralized neuro-embryologic growth gradient, including a “rearward and dorsal” twist of the left hemisphere and a “forward and ventral” twist of the right hemisphere. However, the dorsal-ventral twist was based only on a preliminary observation at the time (Dooling et al. 1983), and our observations of adults in the present study showed an opposite direction, that is, a ventral twist of the left hemisphere, and a dorsal twist of the right. However, the typical adult brain asymmetries are the endpoint of a dynamic developmental process that also plays out through childhood and adolescence, and may involve some reconfiguration (Shaw et al. 2009). Possible mechanisms may include inter-hemispheric differences in neural pruning (Fullard et al. 2019), axon tension (Van Essen 1997), and/or ventricular cerebrospinal fluid volume (Maller et al. 2014) during neural development.

Establishing the genetic contributions to brain asymmetry would help elucidate the developmental origins of this trait, as well as potentially its evolution, and the neural basis of functional lateralization. In the present study, we found heritabilities of 4–13% for the 2 global brain asymmetry measures in the UK Biobank and HCP datasets. This is lower than the 10–30% heritabilities reported in a previous study of vervet monkeys using a similar registration-based approach (Fears et al. 2011), which might suggest a species difference in the degree of genetic control of global brain asymmetry (Xiang et al. 2019).

Our GWAS analyses revealed no loci that surpassed the standard genome-wide significance threshold *P* < 5e−08, for either horizontal or vertical skew. Other recent GWAS studies (Carrion-Castillo et al. 2019a; Le Guen et al. 2020; Sha et al. 2021), also using UK Biobank data, were able to identify significant loci affecting various regional asymmetries (cortical regional surface area and thickness asymmetries, and subcortical volume asymmetries), which had heritabilities in the same range as the skew measures in the present study. In addition, both skews showed high measurement reliability in twice-scanned individuals. The lack of significant GWAS results with the skew measures was therefore likely due to high polygenicity, as was indicated by causal mixture model analysis in the UK Biobank data. This is also consistent with the associations of skew measures with various different regional gray and white matter metrics, many of which can have distinct genetic contributions (Elliott et al. 2018; Grasby et al. 2020), such that the genetic architecture of global asymmetry measures is likely to be particularly complex. Even larger GWAS analyses will be required to pinpoint significant SNPs associated with brain skews.

Our gene-based analysis, in which SNP-level association statistics were combined at the gene-level for a single test per gene, did not identify individual genes at a statistically significant level after adjustment for multiple testing. The most significant gene *PLEC* encodes plectin, which is a cytoskeletal protein linking the 3 main components of the cytoskeleton: actin microfilaments, microtubules and intermediate filaments (Svitkina et al. 1996). Cytoskeletal-related genes have been implicated by other, recent genetic studies of regional structural brain asymmetry and handedness, as well as functional hemispheric language dominance (de Kovel and Francks 2019; Wiberg et al. 2019; Carrion-Castillo et al. 2019b; Cuellar-Partida et al. 2020; Sha et al. 2021). Cytoskeletal-mediated mechanisms for left–right asymmetry development have also been described in invertebrates and frogs (Okumura et al. 2008; Tee et al. 2015; Davison et al. 2016; Inaki et al. 2016; Carrion-Castillo et al. 2019b). We have therefore previously proposed the existence of a human brain-intrinsic mechanism of left–right axis determination, involving cytoskeletal influences on cellular chirality, which may be developmentally distinct from left–right laterality of the visceral organs (Carrion-Castillo et al. 2019b).

In addition, gene-set enrichment analysis of the GWAS results for vertical skew tentatively implicated genes involved in neuron projection guidance. This may relate, for example, to the establishment of interhemispheric connections via the corpus callosum. Consistent with this hypothesis, a recent GWAS analysis of publicly released, imaging-derived phenotypes in the UK Biobank (Smith et al. 2020; https://open.win.ox.ac.uk/ukbiobank/big40/pheweb/, database queried on 1 August 2020) found that corpus callosum measures (e.g., fractional anisotropy of the genu of the corpus callosum, and volume of the anterior part of middle corpus callosum) showed the most significant associations annotated to *PLEC*.

As the heritabilities of both global asymmetry measures were low in our analyses, then nongenetic factors also seem likely to influence them. Early life factors such as birth weight, being part of a multiple birth, and breastfeeding, have been shown to correlate with handedness (de Kovel et al. 2019). In the present study, while we did not find any significant associations between global brain asymmetries and early life factors after correction for multiple testing, 2 nominally significant associations were observed with vertical asymmetry: breastfeeding and birth weight. In addition, country of birth was significant even after correction for multiple testing in phenome-wide association analysis. There may therefore be aspects of prenatal and perinatal behavior, or care, which differ systematically between the countries of the United Kingdom, and can affect brain shape development. Population-genetic differences may also be involved, as may environmental influences later in life.

### Other Notable Phenotypic and Genetic Correlations with Brain Asymmetry Skews

As abnormal brain asymmetry patterns have been reported in a variety of cognitive and neuropsychiatric disorders, including dyslexia (Pieniadz et al. 1983), schizophrenia (Ratnanather et al. 2013), attention-deficit/hyperactivity disorder (Shaw et al. 2009), autism (Eyler et al. 2012), and obsessive–compulsive disorder (Kong et al. 2019), the 2 measures of global asymmetry used in the present study might usefully be analyzed in future studies of these disorders. In the UK Biobank, there were significant phenotypic correlations of both horizontal and vertical brain skew with a depression-related variable, recent changes in speed/amount of moving or speaking. This may be consistent with previous reports of altered occipital bending in major depression (Maller et al. 2014; Fullard et al. 2019). Within the HCP dataset, there was a positive correlation between horizontal skew and oral reading recognition ability. Language-related cognition is well known to make use of lateralized functional networks (Fedorenko and Thompson-Schill 2014; Kong et al. 2020a). In addition, we found evidence for genetic correlation between horizontal brain skew and ASD, which may be consistent with the subtle but widespread alterations of regional cortical thickness asymmetry in ASD reported in a recent, large-scale study (Postema et al. 2019). The automated measurement of global brain asymmetry that we have employed here will be feasible for large-scale meta-analysis-based studies of brain disorders, such as those carried out within consortia such as ENIGMA (http://enigma.ini.usc.edu/; Thompson et al. 2019; Kong et al. 2020c).

In addition, we found horizontal and vertical brain skew measures to show significant phenotypic correlations with BMI in the UK Biobank. This is consistent with a recent observation that BMI correlated with regional thickness of frontal and occipital regions, in opposite directions in the 2 hemispheres, in a study of 895 healthy adults (Vainik et al. 2018). Further, studies will be needed to disentangle any potential cause–effect relations and underlying mechanisms linking these traits. For the pheWAS analysis with brain skew measures, we adjusted for various covariate effects: sex, age, nonlinear age, the first 10 PCs that capture genome-wide population structure in the genotype data, and technical variables related to imaging (imaging assessment center, scanner position parameters, and signal/contrast-to-noise ratio in T1). Nonetheless, other confounding effects may have been relevant for the associations with some phenotypes. PheWAS is a screening approach, applied under a single model with fixed covariates, across thousands of phenotypes. In principle, any one of the thousands of phenotypes could be a relevant confounder for any of the others when assessing its relation with brain skew. There may also be unmeasured, underlying effects that cause some combinations of traits to be associated. For these reasons, we make no claims about cause-effect relations based on the PheWAS results.

## Conclusion

In sum, the present study used automated, registration-based measurement of the horizontal and vertical components of global brain asymmetry, both of which showed high test–retest repeatability. With the largest-ever analyses, we revealed 2 average asymmetry patterns at the population level: one global asymmetry along the anterior–posterior axis, and one along the dorsal-ventral axis. Furthermore, we clarified the relationships between global brain asymmetries and handedness, linking brain structural asymmetries to one of the most clearly evident functional lateralizations, although the effect sizes were small. The 2 asymmetrical skew measures also showed associations with diverse metrics of regional gray matter and white matter, as well as various phenotypic variables related to cognitive functions, sociodemographic and physical factors, and mental health. Genetic analyses indicated low heritability and high polygenicity of the brain skew measures, and a potential genetic overlap with ASD. Together, our results provide evidence for the functional significance of global brain asymmetry, and indicate that genetic variation plays a role—although not a determining one—in its variation in the population.

## Notes

We thank Nathalie Tzourio-Mazoyer for sharing her extensive knowledge of brain laterality. This research was conducted using the UK Biobank resource under application number 16 066, with Clyde Francks as the principal applicant. Our study made use of imaging-derived phenotypes generated by an image-processing pipeline developed and run on behalf of UK Biobank. We thank the UK Biobank and the HCP for data sharing.

*Conflict of Interest*: The authors declare no competing financial interests.

## Funding

This research was funded by the Max Planck Society (Germany) and grants from the Netherlands Organization for Scientific Research (NWO; grant no. 054-15-101) and the French National Research Agency (ANR, grant no. 15-HBPR-0001-03), as part of the FLAG-ERA consortium project “MULTI-LATERAL”, a Partner Project to the European Union’s Flagship Human Brain Project.

## Authors’ Contributions

X.Z.K. and C.F. designed the research. X.Z.K, M.P., A.P., D.S. and A.C.C performed data analysis; F.C., M.J., B.M., S.E.F. and C.F. obtained funding. X.Z.K. and C.F. drafted the paper. All authors provided advice on the study and feedback on the paper.

## Supplementary Material

Kong_skewSM_revision_v6_bhab075

Dataset_S1_bhab075

Dataset_S2_bhab075

## References

[ref1] Alfaro-Almagro F , Jenkinson M, Bangerter NK, Andersson JLR, Griffanti L, Douaud G, Sotiropoulos SN, Jbabdi S, Hernandez-Fernandez M, Vallee E et al. 2018. Image processing and quality control for the first 10,000 brain imaging datasets from UK Biobank. Neuroimage. 166:400–424. 29079522 10.1016/j.neuroimage.2017.10.034 PMC5770339

[ref2] Allen JS , Damasio H, Grabowski TJ, Bruss J, Zhang W. 2003. Sexual dimorphism and asymmetries in the gray-white composition of the human cerebrum. Neuroimage. 18:880–894. 12725764 10.1016/s1053-8119(03)00034-x

[ref3] Almasy L , Blangero J. 1998. Multipoint quantitative-trait linkage analysis in general pedigrees. Am J Hum Genet. 62:1198–1211. 9545414 10.1086/301844 PMC1377101

[ref4] Ashburner M , Ball CA, Blake JA, Botstein D, Butler H, Cherry JM, Davis AP, Dolinski K, Dwight SS, Eppig JT et al. 2000. Gene ontology: tool for the unification of biology. The gene ontology Consortium. Nat Genet. 25(1):25–29. 10802651 10.1038/75556 PMC3037419

[ref5] Balzeau A , Gilissen E, Grimaud-Herve D. 2011. Shared pattern of endocranial shape asymmetries among great apes, anatomically modern humans, and fossil hominins. PLoS One. 7:e29581. 22242147 10.1371/journal.pone.0029581 PMC3252326

[ref6] Barch DM , Burgess GC, Harms MP, Petersen SE, Schlaggar BL, Corbetta M, Glasser MF, Curtiss S, Dixit S, Feldt C et al. 2013. Function in the human connectome: task-fMRI and individual differences in behavior. Neuroimage. 80:169–189. 23684877 10.1016/j.neuroimage.2013.05.033 PMC4011498

[ref7] Barrick TR , Mackay CE, Prima S, Maes F, Vandermeulen D, Crow TJ, Roberts N. 2005. Automatic analysis of cerebral asymmetry: an exploratory study of the relationship between brain torque and planum temporale asymmetry. Neuroimage. 24:678–691. 15652303 10.1016/j.neuroimage.2004.09.003

[ref8] Batista-Garcia-Ramo K , Fernandez-Verdecia CI. 2018. What we know about the brain structure-function relationship. Behav Sci. 8(4):39. 10.3390/bs8040039 PMC5946098 29670045

[ref9] Bear D , Schiff D, Saver J, Greenberg M, Freeman R. 1986. Quantitative analysis of cerebral asymmetries. Fronto-occipital correlation, sexual dimorphism and association with handedness. Arch Neurol. 43:598–603. 3718289 10.1001/archneur.1986.00520060060019

[ref10] Best CT . 1986. The emergence of cerebral asymmetries in early human development: a literature review and a neuroembryological model. In: Segalowitz DLMSJ, editor. Brain lateralization in children: developmental implications. New York, (NY): Guilford Press, pp. 5–34.

[ref11] Bishop DV . 2013. Cerebral asymmetry and language development: cause, correlate, or consequence? Science. 340:1230531. 23766329 10.1126/science.1230531 PMC4031634

[ref12] Bycroft C , Freeman C, Petkova D, Band G, Elliott LT, Sharp K, Motyer A, Vukcevic D, Delaneau O, O'Connell J et al. 2018. The UK Biobank resource with deep phenotyping and genomic data. Nature. 562(7726):203–209. 10.1038/s41586-018-0579-z PMC6786975 30305743

[ref14] Carrion-Castillo A , Pepe A, Kong X-Z, Fisher SE, Mazoyer B, Tzourio-Mazoyer N, Crivello F, Francks C. 2019a. Genetic effects on planum temporale asymmetry and their limited relevance to neurodevelopmental disorders, intelligence or educational attainment. Cortex. 124:137–153. 31887566 10.1016/j.cortex.2019.11.006

[ref15] Carrion-Castillo A , Van der Haegen L, Tzourio-Mazoyer N, Kavaklioglu T, Badillo S, Chavent M, Saracco J, Brysbaert M, Fisher SE, Mazoyer B et al. 2019b. Genome sequencing for rightward hemispheric language dominance. Genes Brain Behav. 18(5):e12572. 30950222 10.1111/gbb.12572 PMC6850193

[ref16] Chapple B , Grech A, Sham P, Toulopoulou T, Walshe M, Schulze K, Morgan K, Murray RM, McDonald C. 2004. Normal cerebral asymmetry in familial and non-familial schizophrenic probands and their unaffected relatives. Schizophr Res. 67:33–40. 14741322 10.1016/s0920-9964(03)00095-1

[ref17] Chiu HC , Damasio AR. 1980. Human cerebral asymmetries evaluated by computed tomography. J Neurol Neurosurg Psychiatry. 43:873–878. 7441265 10.1136/jnnp.43.10.873 PMC490706

[ref18] Crow TJ . 1997. Schizophrenia as failure of hemispheric dominance for language. Trends Neurosci. 20:339–343. 9246721 10.1016/s0166-2236(97)01071-0

[ref19] Cuellar-Partida G , Tung JY, Eriksson N, Albrecht E, Aliev F, Andreassen OA, Barroso I, Beckmann JS, Boks MP, Boomsma DI et al. 2020. Genome-wide association study identifies 48 common genetic variants associated with handedness. Nat Hum Behav. 5:59–70. 32989287 10.1038/s41562-020-00956-y PMC7116623

[ref20] Davison, A., McDowell, G.S., Holden, J.M., Johnson, H.F., Koutsovoulos, G.D., Liu, M.M., Hulpiau, P., Van Roy, F., Wade, C.M., Banerjee, R., 2016. Formin is associated with left-right asymmetry in the pond snail and the frog. Curr Biol 26, 654–660. 26923788 10.1016/j.cub.2015.12.071 PMC4791482

[ref21] de Kovel CG , Lisgo S, Karlebach G, Ju J, Cheng G, Fisher SE, Francks C. 2017. Left-right asymmetry of maturation rates in human embryonic neural development. Biol Psychiatry. 82(3):204–212. 28267988 10.1016/j.biopsych.2017.01.016

[ref22] de Kovel CGF , Carrion-Castillo A, Francks C. 2019. A large-scale population study of early life factors influencing left-handedness. Sci Rep. 9:584. 30679750 10.1038/s41598-018-37423-8 PMC6345846

[ref23] de Kovel CGF , Francks C. 2019. The molecular genetics of hand preference revisited. Sci Rep. 9:5986. 30980028 10.1038/s41598-019-42515-0 PMC6461639

[ref24] de Kovel CGF , Lisgo SN, Fisher SE, Francks C. 2018. Subtle left-right asymmetry of gene expression profiles in embryonic and foetal human brains. Sci Rep. 8:12606. 30181561 10.1038/s41598-018-29496-2 PMC6123426

[ref25] de Leeuw CA , Mooij JM, Heskes T, Posthuma D. 2015. MAGMA: generalized gene-set analysis of GWAS data. PLoS Comput Biol. 11, e1004219. 10.1371/journal.pcbi.1004219 PMC4401657 25885710

[ref27] Demontis D , Walters RK, Martin J, Mattheisen M, Als TD, Agerbo E, Baldursson G, Belliveau R, Bybjerg-Grauholm J, Baekvad-Hansen M et al. 2019. Discovery of the first genome-wide significant risk loci for attention deficit/hyperactivity disorder. Nat Genet. 51(1):63–75. 30478444 10.1038/s41588-018-0269-7 PMC6481311

[ref28] Dooling EC , Chi JG, Gilles FH. 1983. Telencephalic development: changing gyral patterns. In: Gilles FH, Leviton A, Dooling EC, editors. The developing human brain: growth and epidemiologic neuropathology. Boston, MA: John Wright, PSG Inc., pp. 94–104.

[ref29] Dragovic M , Milenkovic S, Kocijancic D, Zlatko S. 2013. Etiological aspect of left-handedness in adolescents. Srp Arh Celok Lek. 141(5–6):354–358. 23858807 10.2298/sarh1306354d

[ref30] Elliott LT , Sharp K, Alfaro-Almagro F, Shi S, Miller KL, Douaud G, Marchini J, Smith SM. 2018. Genome-wide association studies of brain imaging phenotypes in UK Biobank. Nature. 562:210–216. 30305740 10.1038/s41586-018-0571-7 PMC6786974

[ref31] Eyler LT , Pierce K, Courchesne E. 2012. A failure of left temporal cortex to specialize for language is an early emerging and fundamental property of autism. Brain. 135:949–960. 22350062 10.1093/brain/awr364 PMC3286331

[ref32] Fears SC , Scheibel K, Abaryan Z, Lee C, Service SK, Jorgensen MJ, Fairbanks LA, Cantor RM, Freimer NB, Woods RP. 2011. Anatomic brain asymmetry in vervet monkeys. PLoS One. 6:e28243. 22205941 10.1371/journal.pone.0028243 PMC3244392

[ref33] Fedorenko E , Thompson-Schill SL. 2014. Reworking the language network. Trends Cogn Sci. 18:120–126. 24440115 10.1016/j.tics.2013.12.006 PMC4091770

[ref34] Finucane HK , Bulik-Sullivan B, Gusev A, Trynka G, Reshef Y, Loh PR, Anttila V, Xu H, Zang CZ, Farh K et al. 2015. Partitioning heritability by functional annotation using genome-wide association summary statistics. Nat Genet. 47(11):1228. 26414678 10.1038/ng.3404 PMC4626285

[ref35] Francks C . 2015. Exploring human brain lateralization with molecular genetics and genomics. Ann N Y Acad Sci. 1359:1–13. 25950729 10.1111/nyas.12770

[ref36] Fullard K , Maller JJ, Welton T, Lyon M, Gordon E, Koslow SH, Grieve SM. 2019. Is occipital bending a structural biomarker of risk for depression and sensitivity to treatment? J Clin Neurosci. 63:55–61. 30827879 10.1016/j.jocn.2019.02.007

[ref37] Galaburda AM , LeMay M, Kemper TL, Geschwind N. 1978. Right-left asymmetrics in the brain. Science. 199:852–856. 341314 10.1126/science.341314

[ref38] Gazzaniga MS . 2009. Human: the science behind what makes your brain unique. New York, NY: HarperCollins.

[ref39] Glasser MF , Sotiropoulos SN, Wilson JA, Coalson TS, Fischl B, Andersson JL, Xu J, Jbabdi S, Webster M et al. 2013. The minimal preprocessing pipelines for the human connectome project. Neuroimage. 80:105–124. 23668970 10.1016/j.neuroimage.2013.04.127 PMC3720813

[ref40] Good CD , Johnsrude I, Ashburner J, Henson RN, Friston KJ, Frackowiak RS. 2001. Cerebral asymmetry and the effects of sex and handedness on brain structure: a voxel-based morphometric analysis of 465 normal adult human brains. Neuroimage. 14:685–700. 11506541 10.1006/nimg.2001.0857

[ref41] Grasby KL , Jahanshad N, Painter JN, Colodro-Conde L, Bralten J, Hibar DP, Lind PA, Pizzagalli F, Ching CRK, McMahon MAB et al. 2020. The genetic architecture of the human cerebral cortex. Science. 367:eaay6690. 32193296 10.1126/science.aay6690 PMC7295264

[ref42] Grove J , Ripke S, Als TD, Mattheisen M, Walters RK, Won H, Pallesen J, Agerbo E, Andreassen OA, Anney R et al. 2019. Identification of common genetic risk variants for autism spectrum disorder. Nat Genet. 51:431–444. 30804558 10.1038/s41588-019-0344-8 PMC6454898

[ref43] Gunturkun O , Strockens F, Ocklenburg S. 2020. Brain lateralization: a comparative perspective. Physiol Rev. 100:1019–1063. 32233912 10.1152/physrev.00006.2019

[ref44] Heikkila K , Van Beijsterveldt CEM, Haukka J, Iivanainen M, Saari-Kemppainen A, Silventoinen K, Boomsma DI, Yokoyama Y, Vuoksimaa E. 2018. Triplets, birthweight, and handedness. Proc Natl Acad Sci U S A. 115:6076–6081. 29760105 10.1073/pnas.1719567115 PMC6003315

[ref45] Herve PY , Crivello F, Perchey G, Mazoyer B, Tzourio-Mazoyer N. 2006. Handedness and cerebral anatomical asymmetries in young adult males. Neuroimage. 29:1066–1079. 16198126 10.1016/j.neuroimage.2005.08.031

[ref46] Hoggart CJ , Clark TG, De Iorio M, Whittaker JC, Balding DJ. 2008. Genome-wide significance for dense SNP and resequencing data. Genet Epidemiol. 32:179–185. 18200594 10.1002/gepi.20292

[ref47] Holland D , Frei O, Desikan R, Fan CC, Shadrin AA, Smeland OB, Sundar VS, Thompson P, Andreassen OA, Dale AM. 2020. Beyond SNP heritability: polygenicity and discoverability of phenotypes estimated with a univariate Gaussian mixture model. PLoS Genet. 16:e1008612. 32427991 10.1371/journal.pgen.1008612 PMC7272101

[ref48] Holloway RL . 2015. The evolution of the hominid brain. In: Handbook of paleoanthropology. Berlin, Heidelberg: Springer, pp. 1961–1987.

[ref49] Hopkins WD , Taglialatela JP, Meguerditchian A, Nir T, Schenker NM, Sherwood CC. 2008. Gray matter asymmetries in chimpanzees as revealed by voxel-based morphometry. Neuroimage. 42:491–497. 18586523 10.1016/j.neuroimage.2008.05.014 PMC2569890

[ref50] Inaki M , Liu J, Matsuno K. 2016. Cell chirality: its origin and roles in left–right asymmetric development. Philos Trans R Soc B. 371:20150403. 10.1098/rstb.2015.0403 PMC5104503 27821533

[ref51] Jackson H . 1874. On the nature of the duality of the brain. Med Press Circular. 1:80–86.

[ref52] Josse G , Mazoyer B, Crivello F, Tzourio-Mazoyer N. 2003. Left planum temporale: an anatomical marker of left hemispheric specialization for language comprehension. Brain Res Cogn Brain Res. 18:1–14. 14659492 10.1016/j.cogbrainres.2003.08.007

[ref53] Kertesz, A., Geschwind, N., 1971. Patterns of pyramidal decussation and their relationship to handedness. Arch Neurol. 24, 326. 10.1001/archneur.1971.00480340058006 5548452

[ref54] Koff E , Naeser MA, Pieniadz JM, Foundas AL, Levine HL. 1986. Computed tomographic scan hemispheric asymmetries in right- and left-handed male and female subjects. Arch Neurol. 43:487–491. 3964116 10.1001/archneur.1986.00520050059023

[ref55] Kong X-Z , Tzourio-Mazoyer N, Joliot M, Fedorenko E, Liu J, Fisher SE, Francks C. 2020a. Gene expression correlates of the cortical network underlying sentence processing. Neurobiol Lang. 1:77–103. 10.1162/nol_a_00004 PMC9923707 36794006

[ref56] Kong XZ , Boedhoe PSW, Abe Y, Alonso P, Ameis SH, Arnold PD, Assogna F, Baker JT, Batistuzzo MC, Benedetti F et al. 2019. Mapping cortical and subcortical asymmetry in obsessive-compulsive disorder: findings from the ENIGMA Consortium. Biol Psychiatry. 87(12):1022–1034. 31178097 10.1016/j.biopsych.2019.04.022 PMC7094802

[ref57] Kong XZ , Group ELW, Francks C. 2020b. Reproducibility in the absence of selective reporting: an illustration from large-scale brain asymmetry research. Hum Brain Mapp. ahead of print https://doi.org/10.1002/hbm.25154. 10.1002/hbm.25154 PMC8675427 32841457

[ref58] Kong XZ , Mathias SR, Guadalupe T, Group ELW, Glahn DC, Franke B, Crivello F, Tzourio-Mazoyer N, Fisher SE, Thompson PM et al. 2018. Mapping cortical brain asymmetry in 17,141 healthy individuals worldwide via the ENIGMA Consortium. Proc Natl Acad Sci U S A. 115:E5154–E5163. 29764998 10.1073/pnas.1718418115 PMC5984496

[ref59] Kong XZ , Postema MC, Guadalupe T, de Kovel C, Boedhoe PSW, Hoogman M, Mathias SR, van Rooij D, Schijven D, Glahn DC et al. 2020c. Mapping brain asymmetry in health and disease through the ENIGMA consortium. Hum Brain Mapp. ahead of print https://doi.org/10.1002/hbm.25033. 10.1002/hbm.25033 PMC8675409 32420672

[ref60] Le Guen Y , Leroy F, Philippe C, Consortium I, Mangin JF, Dehaene-Lambertz G, Frouin V. 2020. Enhancer locus in ch14q23.1 modulates brain asymmetric temporal regions involved in language processing. Cereb Cortex. 30:5322–5332. 32432689 10.1093/cercor/bhaa112

[ref61] Le May M , Kido DK. 1978. Asymmetries of the cerebral hemispheres on computed tomograms. J Comput Assist Tomogr. 2:471–476. 708500 10.1097/00004728-197809000-00018

[ref62] Lee SH , Yang J, Goddard ME, Visscher PM, Wray NR. 2012. Estimation of pleiotropy between complex diseases using single-nucleotide polymorphism-derived genomic relationships and restricted maximum likelihood. Bioinformatics. 28:2540–2542. 22843982 10.1093/bioinformatics/bts474 PMC3463125

[ref63] LeMay M . 1976. Morphological cerebral asymmetries of modern man, fossil man, and nonhuman primate. Ann N Y Acad Sci. 280:349–366. 827951 10.1111/j.1749-6632.1976.tb25499.x

[ref64] LeMay M . 1977. Asymmetries of the skull and handedness. Phrenology Revisited J Neurol Sci. 32:243–253. 874523 10.1016/0022-510x(77)90239-8

[ref65] LeMay M . 1992. Left-right dissymmetry, handedness. AJNR Am J Neuroradiol. 13:493–504. 1566713 PMC8333206

[ref66] Liu HS , Stufflebeam SM, Sepulcre J, Hedden T, Buckner RL. 2009. Evidence from intrinsic activity that asymmetry of the human brain is controlled by multiple factors. Proc Natl Acad Sci U S A. 106:20499–20503. 19918055 10.1073/pnas.0908073106 PMC2777963

[ref67] Luchins DJ , Meltzer HY. 1983. A blind, controlled study of occipital cerebral asymmetry in schizophrenia. Psychiatry Res. 10:87–95. 6140697 10.1016/0165-1781(83)90107-5

[ref68] Maller JJ , Anderson RJ, Thomson RH, Daskalakis ZJ, Rosenfeld JV, Fitzgerald PB. 2017. Occipital bending in schizophrenia. Aust N Z J Psychiatry. 51:32–41. 27066817 10.1177/0004867416642023

[ref69] Maller JJ , Thomson RHS, Rosenfeld JV, Anderson R, Daskalakis ZJ, Fitzgerald PB. 2014. Occipital bending in depression. Brain. 137:1830–1837. 24740986 10.1093/brain/awu072

[ref70] Mazoyer B , Mellet E, Perchey G, Zago L, Crivello F, Jobard G, Delcroix N, Vigneau M, Leroux G, Petit L et al. 2016. BIL & GIN: a neuroimaging, cognitive, behavioral, and genetic database for the study of human brain lateralization. Neuroimage. 124:1225–1231. 25840118 10.1016/j.neuroimage.2015.02.071

[ref71] Mazoyer B , Zago L, Jobard G, Crivello F, Joliot M, Perchey G, Mellet E, Petit L, Tzourio-Mazoyer N. 2014. Gaussian mixture modeling of hemispheric lateralization for language in a large sample of healthy individuals balanced for handedness. PLoS One. 9:e101165. 24977417 10.1371/journal.pone.0101165 PMC4076312

[ref72] McShane D , Risse GL, Rubens AB. 1984. Cerebral asymmetries on CT scan in three ethnic groups. Int J Neurosci. 23:69–74. 6724817 10.3109/00207458408985346

[ref113] Millard LAC , et al. 2017. Software Application Profile: PHESANT: a tool for performing automated phenome scans in UK Biobank. International Journal of Epidemiology. 10.1093/ije/dyx204 PMC5837456 29040602

[ref73] Mock JR , Zadina JN, Corey DM, Cohen JD, Lemen LC, Foundas AL. 2012. Atypical brain torque in boys with developmental stuttering. Dev Neuropsychol. 37:434–452. 22799762 10.1080/87565641.2012.661816 PMC5537737

[ref74] Narr KL , Bilder RM, Luders E, Thompson PM, Woods RP, Robinson D, Szeszko PR, Dimtcheva T, Gurbani M, Toga AW. 2007. Asymmetries of cortical shape: effects of handedness, sex and schizophrenia. Neuroimage. 34:939–948. 17166743 10.1016/j.neuroimage.2006.08.052 PMC3299195

[ref75] Neubauer S , Gunz P, Scott NA, Hublin JJ, Mitteroecker P. 2020. Evolution of brain lateralization: a shared hominid pattern of endocranial asymmetry is much more variable in humans than in great apes. Sci Adv. 6:eaax9935. 32110727 10.1126/sciadv.aax9935 PMC7021492

[ref76] Ocklenburg S , Schmitz J, Moinfar Z, Moser D, Klose R, Lor S, Kunz G, Tegenthoff M, Faustmann P, Francks C et al. 2017. Epigenetic regulation of lateralized fetal spinal gene expression underlies hemispheric asymmetries. Elife. 6:e22784. 28145864 10.7554/eLife.22784 PMC5295814

[ref77] Okumura T , Utsuno H, Kuroda J, Gittenberger E, Asami T, Matsuno K. 2008. The development and evolution of left-right asymmetry in invertebrates: lessons from drosophila and snails. Dev Dyn. 237:3497–3515. 19035360 10.1002/dvdy.21788

[ref78] Oldfield RC . 1971. The assessment and analysis of handedness: the Edinburgh inventory. Neuropsychologia. 9:97–113. 5146491 10.1016/0028-3932(71)90067-4

[ref79] Panagiotou OA , Ioannidis JP, Genome-Wide Significance P. 2012. What should the genome-wide significance threshold be? Empirical replication of borderline genetic associations. Int J Epidemiol. 41:273–286. 22253303 10.1093/ije/dyr178

[ref80] Papadatou-Pastou M , Ntolka E, Schmitz J, Martin M, Munafo MR, Ocklenburg S, Paracchini S. 2020. Human handedness: a meta-analysis. Psychol Bull. 146:481–524. 32237881 10.1037/bul0000229

[ref81] Peters M , Reimers S, Manning JT. 2006. Hand preference for writing and associations with selected demographic and behavioral variables in 255,100 subjects: the BBC internet study. Brain Cogn. 62:177–189. 16797814 10.1016/j.bandc.2006.04.005

[ref82] Pieniadz JM , Naeser MA, Koff E, Levine HL. 1983. CT scan cerebral hemispheric asymmetry measurements in stroke cases with global aphasia: atypical asymmetries associated with improved recovery. Cortex. 19:371–391. 6641244 10.1016/s0010-9452(83)80007-0

[ref83] Postema, M.C., van Rooij, D., Anagnostou, E., Arango, C., Auzias, G., Behrmann, M., Filho, G.B., Calderoni, S., Calvo, R., Daly, E., et al. 2019. Altered structural brain asymmetry in autism spectrum disorder in a study of 54 datasets. Nat Commun 10, 4958. 31673008 10.1038/s41467-019-13005-8 PMC6823355

[ref84] Price CJ . 2012. A review and synthesis of the first 20 years of PET and fMRI studies of heard speech, spoken language and reading. Neuroimage. 62:816–847. 22584224 10.1016/j.neuroimage.2012.04.062 PMC3398395

[ref85] Ratnanather JT , Poynton CB, Pisano DV, Crocker B, Postell E, Cebron S, Ceyhan E, Honeycutt NA, Mahon PB, Barta PE. 2013. Morphometry of superior temporal gyrus and planum temporale in schizophrenia and psychotic bipolar disorder. Schizophr Res. 150:476–483. 24012458 10.1016/j.schres.2013.08.014 PMC3825771

[ref86] Renteria ME . 2012. Cerebral asymmetry: a quantitative, multifactorial, and plastic brain phenotype. Twin Res Hum Genet. 15:401–413. 22856374 10.1017/thg.2012.13

[ref87] Schizophrenia Working Group of the Psychiatric Genomics, C . 2014. Biological insights from 108 schizophrenia-associated genetic loci. Nature. 511:421–427. 25056061 10.1038/nature13595 PMC4112379

[ref88] Sha Z , Schijven D, Carrion-Castillo A, Joliot M, Mazoyer B, Fisher S, Crivello F, Francks C. 2021. The genetic architecture of structural left-right asymmetry of the human brain Nat Hum Behav, Advance online publication https://www.nature.com/articles/s41562-021-01069-w. 10.1038/s41562-021-01069-w PMC8455338 33723403

[ref89] Shaw P , Lalonde F, Lepage C, Rabin C, Eckstrand K, Sharp W, Greenstein D, Evans A, Giedd JN, Rapoport J. 2009. Development of cortical asymmetry in typically developing children and its disruption in attention-deficit/hyperactivity disorder. Arch Gen Psychiatry. 66:888–896. 19652128 10.1001/archgenpsychiatry.2009.103 PMC2948210

[ref90] Smith GE . 1925. The London skull. Br Med J. 2:853–854. 20772218 10.1136/bmj.2.3384.853 PMC2227665

[ref91] Smith SM , Douaud G, Chen W, Hanayik T, Alfaro-Almagro F, Sharp K, Elliott LT. 2020. Enhanced brain imaging genetics in UK Biobank, bioRxiv. 10.1038/s41593-021-00826-4 PMC7610742 33875891

[ref92] Steele J . 2000. Handedness in past human populations: skeletal markers. Laterality. 5:193–220. 15513142 10.1080/713754380

[ref93] Sudlow C , Gallacher J, Allen N, Beral V, Burton P, Danesh J, Downey P, Elliott P, Green J, Landray M et al. 2015. UK biobank: an open access resource for identifying the causes of a Wide range of complex diseases of middle and old age. PLoS Med. 12(3):e1001779. 25826379 10.1371/journal.pmed.1001779 PMC4380465

[ref94] Svitkina TM , Verkhovsky AB, Borisy GG. 1996. Plectin sidearms mediate interaction of intermediate filaments with microtubules and other components of the cytoskeleton. J Cell Biol. 135:991–1007. 8922382 10.1083/jcb.135.4.991 PMC2133373

[ref95] Tee YH , Shemesh T, Thiagarajan V, Hariadi RF, Anderson KL, Page C, Volkmann N, Hanein D, Sivaramakrishnan S, Kozlov MM. 2015. Cellular chirality arising from the self-organization of the actin cytoskeleton. Nat Cell Biol. 17:445–457. 25799062 10.1038/ncb3137

[ref96] Thompson PM et al. 2019. ENIGMA and global neuroscience: a decade of large-scale studies of the brain in health and disease across 43 countries. Transl Psychiatry. 10:1–28. 10.1038/s41398-020-0705-1 PMC7083923 32198361

[ref97] Toga AW , Thompson PM. 2003. Mapping brain asymmetry. Nat Rev Neurosci. 4:37–48. 12511860 10.1038/nrn1009

[ref98] Tzourio-Mazoyer N , Mazoyer B. 2017. Variations of planum temporale asymmetries with Heschl's Gyri duplications and association with cognitive abilities: MRI investigation of 428 healthy volunteers. Brain Struct Funct. 222:2711–2726. 28164245 10.1007/s00429-017-1367-5

[ref99] Vainik U , Baker TE, Dadar M, Zeighami Y, Michaud A, Zhang Y, Garcia Alanis JC, Misic B, Collins DL, Dagher A. 2018. Neurobehavioral correlates of obesity are largely heritable. Proc Natl Acad Sci U S A. 115:9312–9317. 30154161 10.1073/pnas.1718206115 PMC6140494

[ref100] Van Essen DC . 1997. A tension-based theory of morphogenesis and compact wiring in the central nervous system. Nature. 385:313–318. 9002514 10.1038/385313a0

[ref101] Van Essen DC , Glasser MF, Dierker DL, Harwell J, Coalson T. 2012. Parcellations and hemispheric asymmetries of human cerebral cortex analyzed on surface-based atlases. Cereb Cortex. 22:2241–2262. 22047963 10.1093/cercor/bhr291 PMC3432236

[ref102] Vinkhuyzen AAE , Wray NR, Yang J, Goddard ME, Visscher PM. 2013. Estimation and partition of heritability in human populations using whole-genome analysis methods. Annu Rev Genet. 47 47:75. 23988118 10.1146/annurev-genet-111212-133258 PMC4037293

[ref103] Watanabe K , Taskesen E, van Bochoven A, Posthuma D. 2017. Functional mapping and annotation of genetic associations with FUMA. Nat Commun. 8:1826. 29184056 10.1038/s41467-017-01261-5 PMC5705698

[ref104] Watkins KE , Paus T, Lerch JP, Zijdenbos A, Collins DL, Neelin P, Taylor J, Worsley KJ, Evans AC. 2001. Structural asymmetries in the human brain: a voxel-based statistical analysis of 142 MRI scans. Cereb Cortex. 11:868–877. 11532891 10.1093/cercor/11.9.868

[ref105] Weinberger DR , Luchins DJ, Morihisa J, Wyatt RJ. 1982. Asymmetrical volumes of the right and left frontal and occipital regions of the human brain. Ann Neurol. 11:97–100. 7059134 10.1002/ana.410110118

[ref106] Wiberg A , Ng M, Al Omran Y, Alfaro-Almagro F, McCarthy P, Marchini J, Bennett DL, Smith S, Douaud G, Furniss D. 2019. Handedness, language areas and neuropsychiatric diseases: insights from brain imaging and genetics. Brain. 142:2938–2947. 31504236 10.1093/brain/awz257 PMC6763735

[ref107] Winkler AM , Ridgway GR, Webster MA, Smith SM, Nichols TE. 2014. Permutation inference for the general linear model. Neuroimage. 92:381–397. 24530839 10.1016/j.neuroimage.2014.01.060 PMC4010955

[ref108] Xiang L , Crow T, Roberts N. 2019. Cerebral torque is human specific and unrelated to brain size. Brain Struct Funct. 224:1141–1150. 30635713 10.1007/s00429-018-01818-0 PMC6499874

[ref109] Xiang L , Crow TJ, Hopkins WD, Gong Q, Roberts N. 2018. Human torque is not present in chimpanzee brain. Neuroimage. 165:285–293. 29031530 10.1016/j.neuroimage.2017.10.017

[ref110] Yakovlev PI , Rakic P. 1966. Patterns of decussation of bulbar pyramids and distribution of pyramidal tracts on two sides of the spinal cord. Trans Am Neurol Assoc. 91:366–367.

[ref111] Yang J , Lee SH, Goddard ME, Visscher PM. 2011. GCTA: a tool for genome-wide complex trait analysis. Am J Hum Genet. 88:76–82. 21167468 10.1016/j.ajhg.2010.11.011 PMC3014363

[ref112] Zilles K , Dabringhaus A, Geyer S, Amunts K, Qu M, Schleicher A, Gilissen E, Schlaug G, Steinmetz H. 1996. Structural asymmetries in the human forebrain and the forebrain of non-human primates and rats. Neurosci Biobehav Rev. 20:593–605. 8994198 10.1016/0149-7634(95)00072-0

